# Elucidating the Differentiation Synthesis Mechanisms of Differently Colored Resistance Quinoa Seedings Using Metabolite Profiling and Transcriptome Analysis

**DOI:** 10.3390/metabo13101065

**Published:** 2023-10-10

**Authors:** Junna Liu, Jian Liu, Ping Zhang, Qianchao Wang, Li Li, Heng Xie, Hanxue Li, Hongxin Wang, Shunhe Cheng, Peng Qin

**Affiliations:** 1 College of Agronomy and Biotechnology, Yunnan Agricultural University, Kunming 650201, China; 2021110026@stu.ynau.edu.cn (J.L.); lj12345678l@outlook.com (J.L.); 2021110031@stu.ynau.edu.cn (P.Z.); 2020110028@stu.ynau.edu.cn (Q.W.); 2019210130@stu.ynau.edu.cn (L.L.); 2020210159@stu.ynau.edu.cn (H.X.); 2021210172@stu.ynau.edu.cn (H.L.); 2021240157@stu.ynau.edu.cn (H.W.); 2 Jiangsu Lixiahe District Institute of Agricultural Sciences, Yangzhou 225007, China

**Keywords:** quinoa seedling stage, four resistant cultivars, difference analysis, metabolite profiling, transcriptome

## Abstract

Quinoa (*Chenopodium quinoa* wild.), a dicotyledonous plant native to the Andes, is an increasingly popular pseudograin owing to its high nutritional value, stress resistance capabilities, and gluten-free properties. In this study, we aimed to explore the dynamic changes in different varieties of quinoa at the seedling stage and their regulatory networks. Here, we found that the leaves of quinoa showed obvious coloration after 45 days, and four quinoa seedling types (red, white, yellow, and black) were subjected to ultra-performance liquid chromatography–tandem mass spectrometry (UPLC–MS/MS) and transcriptome sequencing to identify their differentially expressed genes and metabolites. A total of 29 differential metabolites and 19 genes (14 structural and 5 regulatory genes) were identified, and consistent differences were observed in the flavonoid, phenolic acid, and alkaloid metabolites in the different quinoa types. These differential metabolites were significantly enriched in flavonoid and flavonol biosynthesis, flavonoid biosynthesis, and phenylpropanoid biosynthesis pathways. In addition, real-time fluorescence quantitative PCR (RT-qPCR) technology was used to detect the expression of four structural genes involved in the flavonoid biosynthesis pathway and four regulatory genes (interaction network). The results revealed that the structural and regulatory gene transcript levels in the flavonoid pathway were higher in the red quinoa cultivars than in the white, yellow, and black. Additionally, the differences in the leaves of these four quinoa cultivars were mainly due to differences in flavonoid, phenolic acid, and alkaloid accumulation. Our findings provide a basis for understanding the accumulation and coloration mechanisms of flavonoids, phenolic acids, and alkaloids in quinoa seedlings of different colors and also provide a theoretical basis for future investigations.

## 1. Introduction

Quinoa (*Chenopodium quinoa* Willd., 2n = 4X = 36) is an annual dicotyledonous, self-pollinating herbaceous plant belonging to the *Amaranthaceae* family (*Chenopodium*). It is an allotetraploid with alternating single leaves and clustered inflorescence. The leaves are shaped like duck palms, and the leaf margins are divided into full and serrated types [1,2,3,4,5]. Originating in the Andes Mountains of South America, quinoa has been cultivated for 5000–7000 years. Quinoa, known as a “golden grain”, belongs to the Chenopodiaceae family alongside spinach (*Spinacia oleracea* L.) and sugar beet (*Beta vulgaris* L.) [6,7]. It is globally popular owing to its comprehensive nutritional value, high functional value, strong stability, and ecological adaptability [8]. The Food and Agriculture Organization of the United Nations has recognized quinoa as the only single and non-genetically modified crop that can meet the nutritional needs of humans [9,10,11]. Quinoa protein has a balanced amino acid composition and multiple functional activities, such as lowering cholesterol and inhibiting α-glycosidase. Moreover, quinoa is rich in high-quality proteins, healthy oils, saponins, polyphenols, and flavonoids and is, thus, a beneficial addition to a healthy diet [12].

Quinoa plants are also more resistant to abiotic stress than our other traditional staple crops [3,4,13,14], and their leaves have a comprehensive nutritional value similar to quinoa grains. Quinoa leaves contain large amounts of protein, all essential amino acids for humans, and high levels of potassium, manganese, and copper [15,16,17]. Additionally, quinoa leaves and tender stems are rich in different nutrients, such as protein, fat, fiber, essential amino acids, minerals, and vitamins, and are excellent sources of nutrients and health-promoting compounds with a unique balance and high nutrient content [18,19,20,21,22]. Quinoa seeds can be classified as red, white, yellow, or black based on their pigmentation [23]. Most differential metabolites were significantly higher in colored quinoa than in white quinoa, and flavonoids and phenolic acids can act as co-pigments of betaine [24]. To date, approximately 37 flavonoids and 29 phenolic acids have been identified and isolated from quinoa seeds, flour, leaves, and shoots [25]. Phenolic compounds, including phenolic acids, flavonoids, and tannins, constitute the most important secondary metabolites. Research has shown that the phenolic compounds in quinoa have excellent antioxidant activities, and the phenolic acid content depends on the color of the quinoa grains [26,27,28]. Thus, the distribution, content, and antioxidant activity of phenolic compounds varies significantly among quinoa cultivars, tissues, and developmental stages. Deeper-colored quinoa cultivars contain higher phenolic compound contents and stronger antioxidant activities than lighter-colored varieties [29,30,31]. Phenolic acids, such as vanillic acid, gallic acid, ferulic acid, p-hydroxybenzoic acid, and their derivatives, were observed in different colored quinoa genotypes. Their levels showed significant differences between different colored quinoa grains [31,32]. Studies have shown that betaine has a stronger antioxidant capacity than phenolic compounds, and the accumulation of betaine and betanin contributes to color formation in both the peel and flesh [33,34]. Betaine has powerful antioxidant, anticancer, hepatoprotective, antibacterial, and anti-inflammatory activities, along with intestinal and immunomodulatory effects. In addition, betaine is hepatoprotective, protects cells from peroxidation and DNA damage, and exhibits anticancer properties [35,36,37,38,39,40]. Therefore, quinoa seedlings are promising value-added vegetables that could help address malnutrition and contribute to food and nutritional security.

Metabolomics is an effective methodology used to analyze the dynamics of metabolites exposed to endogenous or exogenous factors or stimuli. Metabolites are the final products of gene expression in cells, and the transcriptome is a vector of gene expression. Joint analysis of multi-omics data is more conducive to the study of phenotypes and biological process regulation mechanisms in biological models [41,42,43]. The release of quinoa genome sequences has provided the most valuable resource for molecular research, and integrated omics research, including transcriptome and metabolomic analysis, has also been rapidly developed [44,45,46]. However, there are only a few studies on the large-scale detection, identification, and quantification of the chemical and nutritional components in the different varieties of quinoa during the seedling stage.

In this study, we aimed to explore the dynamic changes in different varieties of quinoa at the seedling stage and their regulatory networks. To address this, four independently selected varieties of quinoa at the seedling stage (red, white, yellow, and black) were used as experimental materials to carry out correlation analyses between metabolite profiles and transcriptomes of different quinoa leaf cultivars, with a focus on the differential metabolites of quinoa flavonoid, flavone, flavonol, and phenylpropanoid biosynthesis. This study not only expands our understanding of the complex process of biosynthesis in different cultivars of quinoa seedlings at the metabolic and molecular levels but also provides valuable information to aid in the development of new quinoa-based health products.

## 2. Materials and Methods

### 2.1. Plant Materials and Sampling

**Figure 1:**
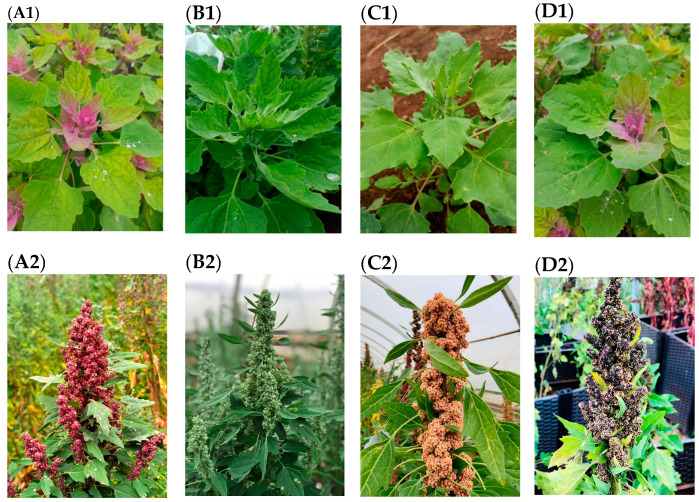

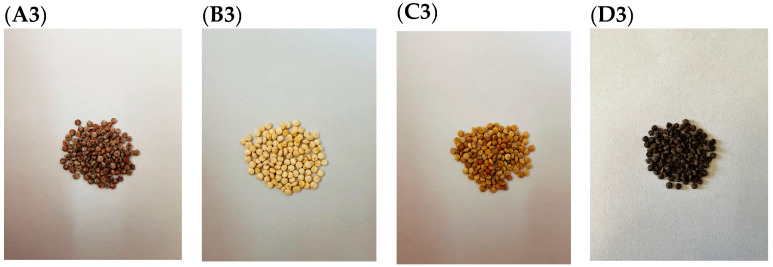
(**A1**–**D1**): Differences in seedling cultivars of red, white, black, and yellow quinoa; (**A2**–**D2**): Differences in grouting period cultivars of red, white, black, and yellow quinoa; (**A3**–**D3**): Differences in seed maturity cultivars of red, white, black, and yellow quinoa.

In this study, four color quinoa seedlings, Dianli-52-3 (R–R, R), Dianli-2019130 (R–W, W), Dianli-QA13-8 (R–Y, Y), and Dianli-Qinghai Black (R–B, B), which were independently selected by Yunnan Agricultural University, were used as test materials. They were planted in a greenhouse at the modern agricultural education base of Yunnan Agricultural University, Xundian County, Kunming City, Yunnan Province (E 102°41′, N 25°20′) with regular water management and normal fertilizer (15 t/hm^2^ of organic fertilizer and 0.75 t/hm^2^ of compound fertilizer [urea (containing N 46%); diammonium phosphate (containing P_2_O_5_ 46%): potassium sulfate (containing K_2_O 40%) = 1:1:0.2]) under identical management conditions for the same period of geographical adaptation observation (Figure 1, Table 1). After 45 d of quinoa planting, quinoa leaves were significantly colored, four cultivars were single-tagged on the same day, and three independent plant leaves with consistent growth were selected from each cultivar (three biological replicates). The samples were immediately frozen in liquid nitrogen and stored at −80 °C until further use.

### 2.2. Morphological Data Collection

Samples were taken from R–R, R–W, R–Y, and R–B seedlings (three replicates) to determine the plant height, stem diameter, leaf area, and chlorophyll content. The height of the quinoa seedlings was measured using vernier calipers (distance from the base to the top of the unfolded leaf). The leaf area was measured using the crop leaf morphology measuring instrument and TPYX-A (https://www.tpyn.net; Shenzhen, China, accessed on 10 August 2023); the TYS-4 N (https://www.tpyn.net; Hangzhou, China, accessed on 10 August 2023) Chlorophyll meter measures chlorophyll. First, we clamped the measuring head of the chlorophyll measuring instrument onto the leaves of the plant to ensure close contact between the measuring head and the leaves. We started the chlorophyll meter, waited for 10 s, and then the chlorophyll meter started measuring the chlorophyll content in the leaves enclosed in the measuring head (this was repeated 3 times). Quinoa leaves were dried by incubating them at 110 °C for 30 min, and they were then dried to a constant weight at 85 °C to determine their physiological indicators.

### 2.3. Differences in Physiological Indicators of Quinoa Cultivars during Seedling Stage

A total alkaloid assay kit [Jiangsu Addison Biotechnology Co., Ltd. (http://www.adsbio.cn, Jiangyin, China, accessed on 10 August 2023) as well as flavonoid, soluble sugar, total amino acid, peroxidase, total phenol, polyphenol oxidase, and phenylalanine aminolase assay kits [Nanjing Jiancheng Institute of Biological Engineering (http://www.njjcbio.com, Nanjing, China, accessed on 10 August 2023) were used. This experimental strictly followed the manufacturer’s instructions (Table S1)].

### 2.4. Metabolite Extraction Detection and Qualitative and Quantitative Analysis

The experimental methods were provided by Wuhan Metware Biotechnology Co., Ltd. (www.metware.cn, Wuhan, China, accessed on 10 August 2023) as previously described [47]. After vacuum freeze-drying (SCIENTZ-100F; Ningbo Scientz Biotechnology Co., Ltd., Ningbo, China), leaves of different colors of quinoa were crushed using a grinding machine (MM400; Retsch GmbH, Haan, Germany) at 30 Hz for 1.5 min, until the material became a powder. The powder (100 mg) was weighed and dissolved in 1.2 mL of 70% methanol solution. It was then vortexed once every 30 min for 30 s, six times. After mixing, the samples were refrigerated at 4 °C. After centrifugation (ANPEL, Shanghai, China) at 12,000 rpm for 10 min, the supernatant was then extracted, and the sample was filtered using a microporous filter membrane (0.22 µm pore size) and then stored in an injection bottle. The extracts were analyzed using ultra-performance liquid chromatography–mass spectrometry (UPLC–MS/MS); the data acquisition instrument system comprises an ultraperformance liquid chromatograph (Nexera X2; Shimadzu, Kyoto, Japan) used with tandem mass spectrometry (MS) (QTRAP^®^ 4500 LC-MS/MS System; Applied Biosystems, Waltham, MA, USA), which accurately quantified the metabolites in the sample. 

Metabolite identification annotations were determined using the Self-built database MWDB (metal database). Process mass spectrometry data used software Analyst 1.6.3. Qualitative analysis of the substances was carried out using secondary spectral information, and metabolite quantification was performed using the multiple reaction monitoring (MRM) mode of a triple quadrupole mass spectrometer. Sample offline mass spectrum files were opened using MultiaQuant 3.0.3 to calibrate the mass spectrum peaks for each metabolite detected in different samples to ensure qualitative and quantitative accuracy. Quality control (QC) samples were used during instrumental analysis to determine the technical repeatability of metabolite extraction and detection. Principal component analysis (PCA) was used to preliminarily understand the overall metabolic differences between each group of samples and the magnitude of variability between samples within the group. By normalizing the metabolite data for the samples using unit variance scaling (UV), cluster heatmap analysis could be performed on all samples. Based on the orthogonal partial least squares discriminant analysis (OPLS-DA) model, the metabolomic data was analyzed, and score maps and permutations for each group were derived, which further demonstrated the differences between each group [48]. Further analyses of differential metabolites with variable importance in projection (VIP) ≥ 1, fold change ≥ 2, and fold change ≤ 0.5 were conducted. Differences were identified using the KEGG database (http://www.kegg.jp/kegg/compound/, accessed on 10 August 2023) and were then annotated, and the differential metabolites were displayed using the KEGG database [49].

### 2.5. Transcriptome Sequencing and Data Analysis

#### 2.5.1. RNA Extraction, Library Construction, and Sequencing

Transcriptome sequencing involves RNA extraction and detection, library construction, and computer sequencing. RNA integrity was assessed using a Fragment Analyzer 5400 (Agilent Technologies, Santa Clara, CA, USA). Ribosomal RNA was removed from the total RNA to obtain mRNA. Subsequently, a fragmentation buffer was added to break the RNA into short segments. Using short-segment RNA as a template, first-strand cDNA was synthesized using six random-base primers. Buffer, dNTPs, and DNA polymerase I were then added to synthesize the second cDNA strand. Subsequently, the double-stranded cDNA was purified using AMPure XP beans. The purified double-stranded cDNA was subjected to end repair, A-tail addition, and sequencing. AMPure XP beads were used for fragment size selection, and PCR enrichment was performed to obtain the final cDNA library. Preliminary quantification was performed using Qubit 2.0 (Life Technologies, Carlsbad, CA, USA), and the insert size of the library was detected using Agilent 2100 (Agilent, Santa Clara, CA, USA). The insert size met the required expectations. The qPCR method accurately quantified the effective concentration of the library (>2 nM), thus completing the library inspection. After passing library inspection, different libraries were pooled according to the target offline data volume and sequenced using the Illumina HiSeq platform (Illumina, San Diego, CA, USA).

#### 2.5.2. Analysis of RNA-Seq Data

The data were filtered to obtain clean data; sequence alignment was performed with the specified reference genome (https://www.ncbi.nlm.nih.gov/genome/?Term=Chenopodium+quinoa+Willd, accessed on 10 August 2023) using HISAT2 (v.2.1.0, https://daehwankimlab.github.io/hisat2/, accessed on 10 August 2023) software, and HISAT2 and StringTie v1.3.4 were used to obtain single genes. Functional annotation of unigenes was performed by searching various databases, including NR, Swiss Prot, GO, COG, KOG, Pfam, and KEGG, using BLAST. The expression values were calculated for all genes and normalized to fragments per kilobase of transcript per million mapped fragments (FPKM). All the DEGs among samples were identified using the DESeq package [50]. An FDR (false discovery rate) value of <0.05 and |log_2_ (fold change)| of ≥1 were used as the thresholds for significant differential expression. The identified DEGs were further subjected to enrichment analysis using Gene Ontology (GO) annotations, clusters of orthologous groups of proteins (COGs), and Kyoto Encyclopedia of Genes and Genomes (KEGG) pathway analysis.

### 2.6. Real-Time Fluorescence Quantitative PCR Analysis

RNA was extracted from the R, W, Y, and B cells and was used for RT-qPCR analysis. To verify the reliability of the transcriptome sequencing results, all samples with highly expressed genes were selected for RT-qPCR experiments. We investigated the gene sequences using the https://www.ncbi.nlm.nih.gov/website, accessed on 10 August 2023. The TUB-6 gene was selected as the internal reference gene, and primers for related genes used for RT-qPCR analysis were designed using Beacon Designer 7.9. PerfectStart SYBR qPCR Supermix (TransGen Biotech, Beijing, China) was used for RT-qPCR according to the manufacturer’s instructions, using a StepOnePlus instrument (Applied Biosystems, Foster City, CA, USA). The reaction volume contained 20 µL Perfectstarttm SYBR qPCR Supermix, 0.4 µL calibration solution, 6.8 µL nuclease-free water (RNase-free water), 0.4 µL forward primer, 0.4 µL reverse primer, and 2 µL cDNA (200 µg/µL). The thermal cycle was set as follows: 94 °C (30 s), 94 °C (5 s), and 60 °C (30 s), for 40 cycles. The relative gene expression level was calculated using the 2^−∆∆CT^ method [51]. Finally, we used SPSS 22.0 software for the statistical analysis of differences between RT-qPCR and RNA-seq data results.

### 2.7. Association Analysis of Metabolite Profiling and Transcriptome

The differential genes and metabolites of the same grouping were simultaneously mapped onto the KEGG pathway map, and bar graphs were drawn based on the results of the differential metabolite and gene enrichment analyses, showing the enrichment level of the pathway with both differential metabolites and genes. Correlation analysis was performed for the genes and metabolites detected in each differential grouping, and the Pearson correlation coefficients (PCC) of the genes and metabolites were calculated using the cor program in R. Gene metabolites with Pearson correlation coefficients > 0.8 in each differential grouping were selected to create network plots to represent the correlation between metabolites and genes. The overall correlation between metabolites and genes was obtained using the canonical correlation analysis (CCA) [52] method, which reflects the overall correlation between the two groups of indicators. The O2PLS [53] model was integrated to analyze the overall impact of the metabolomic and transcriptomic data. Key metabolic pathways, genes, and metabolites were selected for subsequent in-depth experimental analyses.

### 2.8. Statistical Analysis

Each of the compounds was measured in triplicate, and the results were analyzed as mean ± standard deviation (SD). Data analyses were performed with SPSS software (version 22.0, SPSS Inc., Chicago, IL, USA). All the functions were implemented in the R software (version 3.4.4, R Core Team, Vienna, Austria).

## 3. Results

### 3.1. Differences in Metabolite Content during the Seedling Stage of Different Quinoa Cultivars

**Figure 2:**
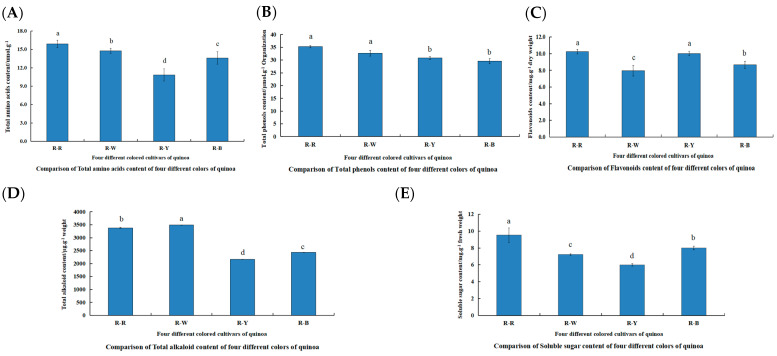
Comparison of physiological indicators between and within R–R, R–W, R–Y, and R–B (**A**–**E**). (**A**): Comparison of total amino acid content of four different colors of quinoa; (**B**): comparison of total phenol content of four different colors of quinoa; (**C**): comparison of flavonoid content of four different colors of quinoa; (**D**): comparison of total alkaloid content of four different colors of quinoa; and (**E**): comparison of soluble sugar content of four different colors of quinoa. Different lowercase letters denote a significant difference at the 0.05 level (*p* < 0.05).

The differences in the physiological indices of R–R, R–W, R–Y, and R–B for total alkaloids, flavonoids, soluble sugars, total amino acids, and total phenols 45 d after planting were compared (Figure 2, Table S1). The content of various indicators in the different quinoa seedling colors were as follows: total amino acids (R–R > R–W > R–B > R–Y), total phenol (R–R > R–W > R–Y > R–B), flavonoids (R–R > R–Y > R–B > R–W), total alkaloids (R–W > R–R > R–B > R–Y), and soluble sugars (R–R > R–B > R–W > R–Y). These samples were used for further metabolite profiling and transcriptome analyses. 

### 3.2. Qualitative and Quantitative Analysis of Metabolites in Different Quinoa Seedling Cultivars

**Figure 3:**
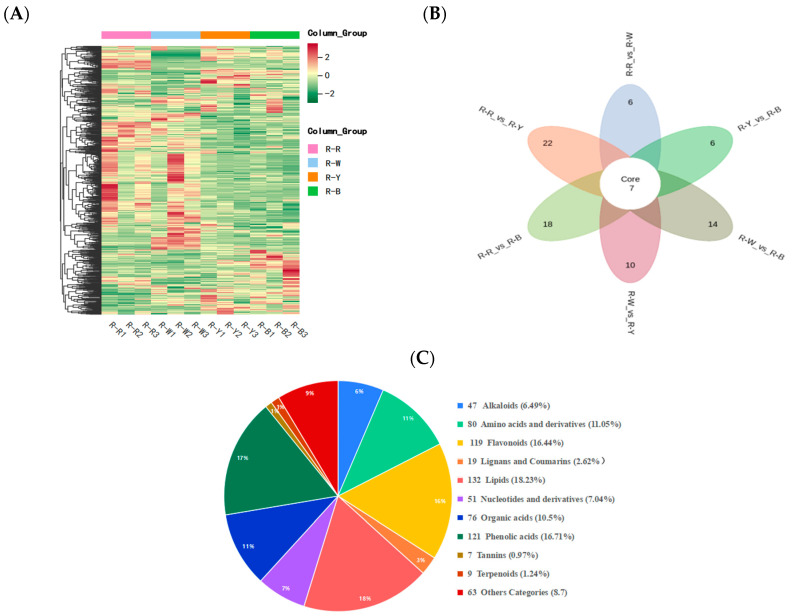
(**A**): Cluster heat map showing differential metabolite accumulations; (**B**): Venn diagram showing the differences between groups; and (**C**): component analysis of the identified metabolites, including type, quantity, and proportion.

Metabolite quantification was accomplished using the multiple reaction monitoring (MRM, Figure S1A,B) mode of triple quadrupole mass spectrometry with accurate and reproducible quantification. By overlapping the total ion flow diagrams of different quality control samples that were detected and analyzed using mass spectrometry, the curve overlap appeared to be high, indicating good instrument stability and technical repeatability, thereby providing important guarantees for the authenticity and reliability of the data (Figure S2A,B). The principal component analysis (Figure S3) and cluster heatmap analysis (Figure 3A) showed that the repeatability within multiple groups of samples was good. In summary, the data generated in this study is of high quality and reliable. Four types of samples, R–R, R–W, R–Y, and R–B, were qualitatively and quantitatively analyzed using a UPLC–MS/MS detection platform and a self-built database. The results showed that 12 samples were selected for this project, with three biological replicates in each group, and in total, 724 metabolites were identified (Figure 3C, Table S2). R–Rvs.R–W, R–Rvs.R–Y, R–Rvs.R–B, R–Wvs.R–Y, R–Wvs.R–B, and R–Yvs.R–B were detected for 717, 722, 722, 719, 722, and 723 metabolites, respectively (Tables S3–S8). Of these, 169, 249, 272, 250, 273, and 112 differential metabolites were detected and quantified (Tables S9–S14) and mainly divided into 10 categories: Amino acids and derivatives, phenolic acids, flavonoids, alkaloids, nucleotides and derivatives, organic acids, lipids, lignans and coumarins, tannins, and terpenoids. Among these, phenolic acids, flavonoids, and alkaloids accounted for a relatively large proportion with significant differences (Figure 3C, Table 2).

### 3.3. Differential Analysis and Enrichment of Metabolites in Different Quinoa Seedling Cultivars

Before screening for differential metabolites, principal component analysis (PCA) was performed on the grouped samples for differential comparison to extract the main information. The results showed a clear separation trend between the groups, with differences between the sample groups. The repeatability of the samples within each group was good (Figure S3). Orthogonal partial least squares discriminant analysis (OPLS-DA) was performed to effectively handle variables with weak correlations. All three groups of OPLS-DA validation plots showed (Figure S4) that the R2Y and Q2 values (in addition to Q^2^ values of R–Wvs.R–Y and R–Yvs.R–B) were greater than 0.9 (*p* < 0.05), indicating that the proposed model was stable and reliable. Based on the OPLS-DA results, a combination of fold change and variable importance in projection (VIP) values of the OPLS-DA model was adopted to screen the differential metabolites. Differential metabolites with VIP ≥ 1, fold change ≥ 2, and fold change ≤ 0.5 were selected. The clustering heat map of the different metabolites (Figure 3A) showed that there were significant differences in the quinoa seedlings among the different strains, with 11 categories, among which phenolic acids, flavonoids, and alkaloids were closely related to quinoa seedling color formation (Table 2). Each point in the differential metabolite volcano plot (Figure S5) represents a metabolite: 169 (61 up and 108 downregulated), 249 (76 up and 173 downregulated), 272 (78 up and 194 downregulated), 250 (97 up and 153 downregulated), 273 (111 up and 162 downregulated) and 112 (50 up and 62 downregulated) metabolites were significantly different (Table 1 and Tables S9–S14). We speculated that these differential metabolites were the main influencing metabolites in the different quinoa cultivars at the seedling stage. Subsequently, the relationship between the differential metabolites in each group was shown in the form of Venn diagrams (Figure 3B and Figure S6). The results showed that there were seven differential metabolites and other group-specific differential metabolites in all comparison groups (Table S15). The 3-O-acetylpinobanksin, anthranilic acid, acacetin, apigenin-7-O-neohesperidoside, indole 3-acetic acid (IAA), sinapyl alcohol, p-coumaric acid, 2-amino-3-methoxybenzoic acid, phloretin-2′-O-glucoside, and nine unique differential metabolites were annotated into the pathway (Table 3).

**Figure 4:**
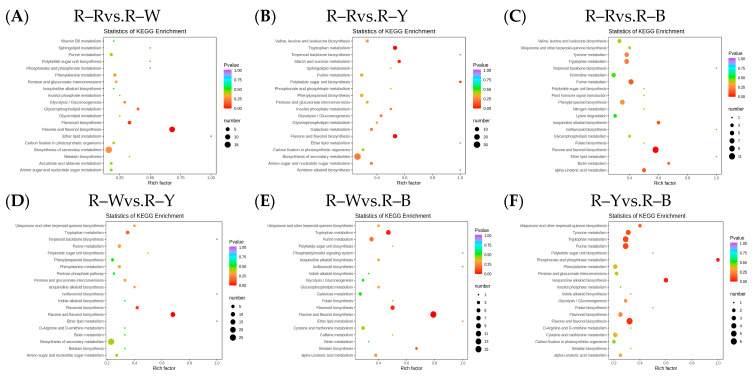
(**A**–**F**): Differential metabolite KEGG enrichment map in different groups. Note: The horizontal axis represents the rich factor corresponding to each pathway, the vertical axis represents the pathway name, and the color of the point is the *p*-value. The redder the point, the more significant the enrichment. The size of the point represents the number of enriched differential metabolites.

After qualitative and quantitative analyses of the detected metabolites, the multiple differences in the quantitative information among the six groups were compared. The differential ploidy histogram (Figure S7) and differential metabolite KEGG enrichment plot (Figure 4A–F) revealed that the differential metabolites that varied significantly between the six groups included flavonoids, phenolic acids, amino acids and their derivatives, and lipids. Differential metabolites interact within organisms to form different pathways. Of the 108, 173, 194, 153, 162, and 62 constitutively upregulated metabolites in the R–R, R–W, R–Y, and R–B treatments, respectively, only 43, 73, 83, 68, 74, and 47 differentially accumulated metabolites were annotated in the pathway (Tables S9–S14). The KEGG enrichment map of the differential metabolites showed that flavonoid biosynthesis was significantly enriched in R–Rvs.R–W (four DAMs), R–Wvs.R–Y (five DAMs), and R–Wvs.R–B (six DAMs), whereas flavone and flavonol biosynthesis were significantly enriched in R–Rvs.R–Y (10), R–Rvs.R–B (11), and R–Yvs.R–B (six), and tryptophan metabolism was only enriched in R–Rvs.R–Y (nine) and R–Wvs.R–B (six). R–B (eight) was significantly enriched, whereas isoquinoline alkaloid biosynthesis and phosphonate and phosphonate metabolism were only significantly enriched in R–Yvs.R–B (three and two differential metabolites) (Figure 4A–F, Table 4). The different cultivars of quinoa seedlings possibly contained flavonoids, phenolic acids, alkaloids, flavonoids, flavones, and flavonols. 

### 3.4. Transcriptome Analysis of Quinoa Seedlings among Different Cultivars

**Figure 5:**
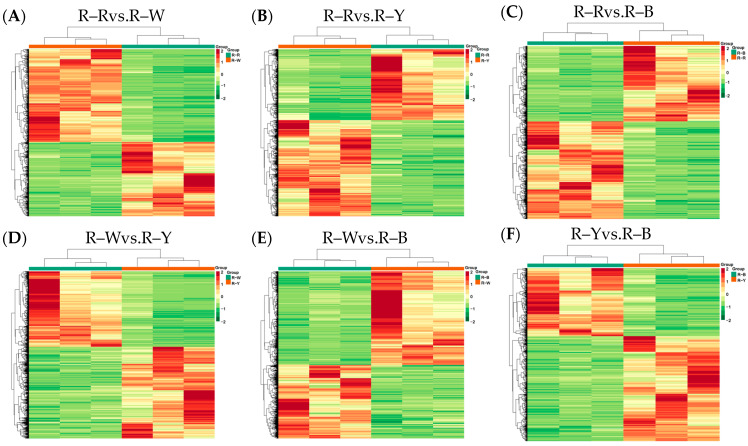
(**A**–**F**): Heat map showing differential gene clustering in different groups. Note: Horizontal coordinates indicate sample names and hierarchical clustering results; vertical coordinates indicate differential gene expression and hierarchical clustering results. Red indicates high expression, and green indicates low expression.

Next, we sequenced and analyzed the transcriptomes of four differently colored quinoa seedlings (three biological repeats for each variety). Raw data was filtered, sequencing error and GC content distribution were checked, 12 samples were sequenced and analyzed, and 156.02 Gb of clean data was obtained overall. The clean data for all samples reached 6 Gb; the percentage of Q30 bases was ≥92%, and the GC content was ≥43.0% (Table S3). The proportion of sequencing reads that successfully matched the genome was >70%, and the matching efficiency was >90%. The efficiency of comparison between the transcriptome data and reference genome was high (>70%), which indicated that the reference genome was well assembled, and the transcriptome data showed a high level of consistency with the reference genome. This indicated that the sequencing results were accurate and could be analyzed in subsequent steps. Pearson’s correlation coefficient (r) was used as an index to assess the correlation of biological replicates; |r| values closer to 1 indicated a stronger correlation between the samples. The correlation coefficients of gene expression levels between biological replicates for all samples were >0.8, and a clear separation was observed between samples, indicating good biological replication and differences between samples of different species. Principal component analysis (PCA) plots (Figure S8), with three replicates of each group of samples, were clustered together, and this indicated that the data was of high quality and showed good stability throughout the method. Gene expression levels of the FPKM values spanned between log10^−2^–log10^4^. The centralized and normalized FPKM expression levels of the differential genes were extracted, a hierarchical cluster analysis was performed, and a cluster heatmap of each differential group was drawn (Figure 5), which clearly showed the differences in gene expression between groups.

### 3.5. Differential Gene Expression Analysis of the Transcriptomes from Different Quinoa Seedling Cultivars

Functional annotation was performed on the detected genes using the KEGG, GO, NR, SwissProt, KOG, Pfam, and Tremble databases. Functionally annotated genes were reported with KEGG (38,122), GO (38,862), NR (49,088), SwissProt (32,844), KOG (46,755), Pfam (42,765), and Tremble (47,789) analyses and involved 142 pathways. Differentially expressed gene analysis was completed using DESeq2, based on the pairwise comparison with a |log_2_ (fold change)| of ≥1 and FDR value of <0.05 as the threshold. The results showed that R–Rvs.R–W, R–Rvs.R–Y, R–Rvs.R–B, R–Wvs.R–Y, R–Wvs.R–B, and R–Yvs.R–B detected 2370 (1052 up- and 1318 downregulated genes in R–R), 4507 (1915 up- and 2592 downregulated genes in R–R), 3125 (1365 up- and 1760 downregulated genes in R–R), 6473 (2927 up- and 3547 downregulated genes in R–W), 4436 (2487 up- and 1949 downregulated genes in R–W), and 1492 (905 up- and 587 downregulated genes in R–Y) genes (Table 5, Tables S16–S21), and MA plots were used to visualize the overall distribution of the gene expression levels and differential ploidy (Figure S9). The FPKM expression, after extracting the differential genes centrally and normalizing and performing hierarchical clustering analysis of the clustering heat map, shows that the clustering results differed among the different varieties of differential genes with high expression levels (Figure 5). 

**Figure 6:**
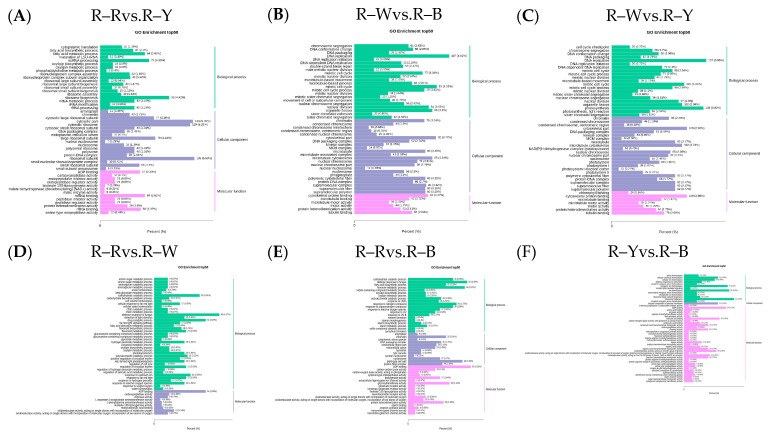
(**A**–**F**): GO enrichment analysis of the differential genes in different groups. Note: The horizontal axis represents the proportion of the annotated genes to the total number of annotated genes, while the vertical axis represents the name of the GO entry. The label on the right side of the graph represents the classifications to which the GO items belong.

**Figure 7:**
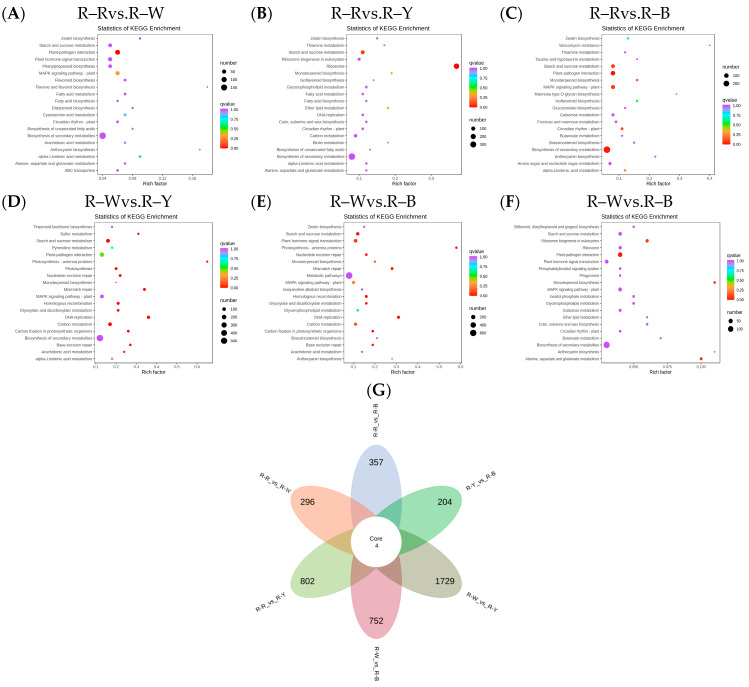
(**A**–**F**): Enrichment scatter plot in different groups; (**G**): differential genetic Venn diagram. Note: Vertical coordinates represent KEGG pathways. The horizontal coordinate indicates the rich factor; the larger the rich factor, the greater the enrichment. A larger dot indicates a greater number of DEGs in the pathway. The redder the color of the dot, the more significant the enrichment. The non-overlapping areas represent the differential genes specific to the differential subgroup, and the overlapping areas represent the differential genes common to several different subgroups.

The Venn diagram shows the overlap of differential genes among the different comparative combinations, and the differential genes that were common or unique to certain comparative combinations can be screened using different groupings of differential gene Venn diagrams (Figure S10). To understand the biological functions of the DEGs, GO (Gene Ontology) term enrichment was performed using BLAST-GO. The results identified 2370 (R–Rvs.R–W), 4507 (R–Rvs.R–Y), 3125 (R–Rvs.R–B), 6473 (R–Wvs.R–Y), 4436 (R–Wvs.R–B), and 1492 (R–Yvs.R–B) DEGs, which were divided into 59 functional groups, including 28 biological process categories, 18 cellular component categories, and 13 molecular function categories (Figure 6A–F). The six comparison groups were shown to have different percentages of the three components, which may also be a key factor in regulating the genetic differences between multiple groups of different quinoa seedling stages. To further identify the metabolic pathways associated with the DEGs, KEGG enrichment analysis was performed. Four common differential genes, 296 (R–Rvs.R–W), 802 (R–Rvs.R–Y), 357 (R–Rvs.R–B), 1729 (R–Wvs.R–Y), 752 (R–Wvs.R–B), and 204 (R–Yvs.R–B) DEGs, were assigned to 126, 131, 134, 136, 134, and 109 KEGG pathways, respectively (Figure 7A–G). Among these, 11, 10, 17, 22, 25, and 20 pathways were significantly enriched (*p* < 0.05, Tables S16–S21). A scatter plot was generated to provide a graphical presentation of the results of the KEGG enrichment analysis, notably starch and sucrose metabolism, plant-pathogen interactions, alpha-linolenic acid metabolism, phenylpropanoid biosynthesis, flavonoid biosynthesis, and anthocyanin biosynthesis in the six comparison groups were significantly enriched to different degrees (Figure 7A–F). In addition, these enriched pathways were further classified into five categories: cellular processes, environmental information processing, genetic information processing, metabolism, and organism systems. Among the five categories, the metabolism category contained the largest number of pathways in all three comparison groups (Figure S11). New transcript information was extracted from the comparison results of the spliced transcripts with genome annotations, and new gene functions were annotated with 4368 new genes that required mining (Table S22). 

### 3.6. Real-Time Fluorescence Quantitative PCR Validation

**Figure 8:**
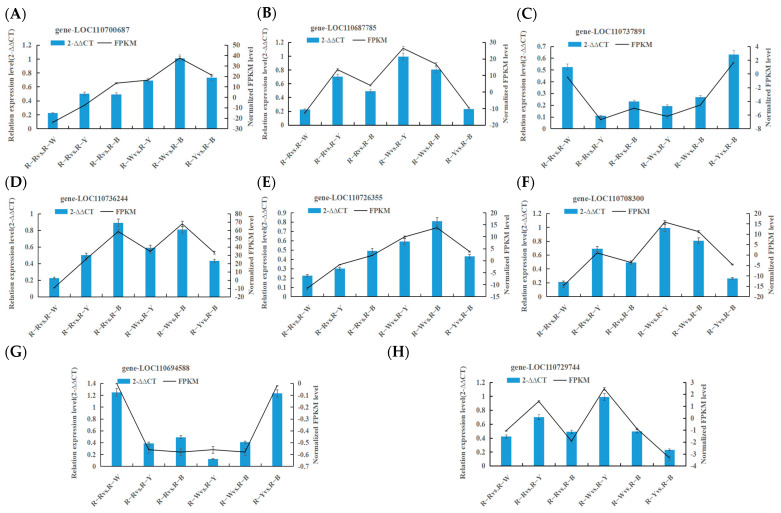
(**A**–**H**): Verifying the transcription level of selected DEGs by RT-qPCR.

Confirmation of DEGs related to key biosynthetic pathways was performed using RT-qPCR with three replicates of each reaction, and 2^−∆∆CT^ was used to analyze the normalized expression of each sample. In this way, we can calculate the 2^−∆∆CT^ and SD and simultaneously calculate the FPKM and SD of the validated genes. Based on the 2^−∆∆CT^ of the validated genes and the FPKM of the sequenced genes, as shown in Figure 8, most selected genes displayed a high correlation between RT-qPCR and RNA-seq datasets, thus verifying the transcriptomic data (Figure 8, Table 6).

### 3.7. Correlation Analysis of the Metabolome and Transcriptomes of Different Quinoa Seedling Cultivars

**Figure 9:**
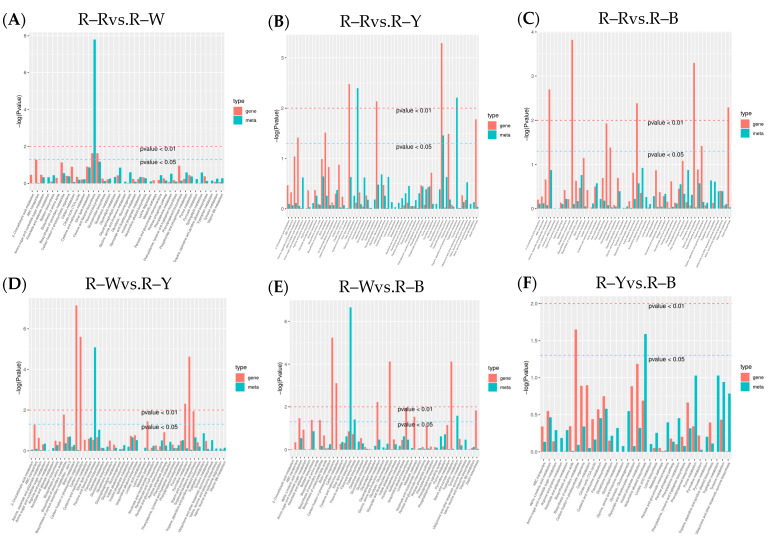
(**A**–**F**): KEGG enrichment analysis in different groups. Note: The horizontal coordinates of the bar graph represent the metabolic pathway, the red color in the vertical coordinates represents the enrichment *p*-values for the differential genes, and the green color represents the enrichment *p*-values for the differential metabolites, which are represented by −log (*p*-value). The higher the vertical coordinate, the stronger the enrichment.

**Figure 10:**
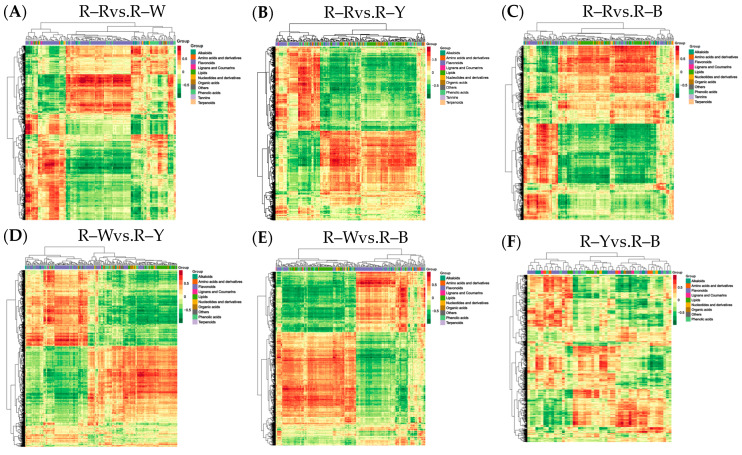
(**A**–**F**): Correlation coefficient clustering heat map in different groups. Note: For differential metabolites with a correlation coefficient above 0.8, select all the correlation calculation results and draw the correlation coefficient cluster heat map.

**Figure 11:**
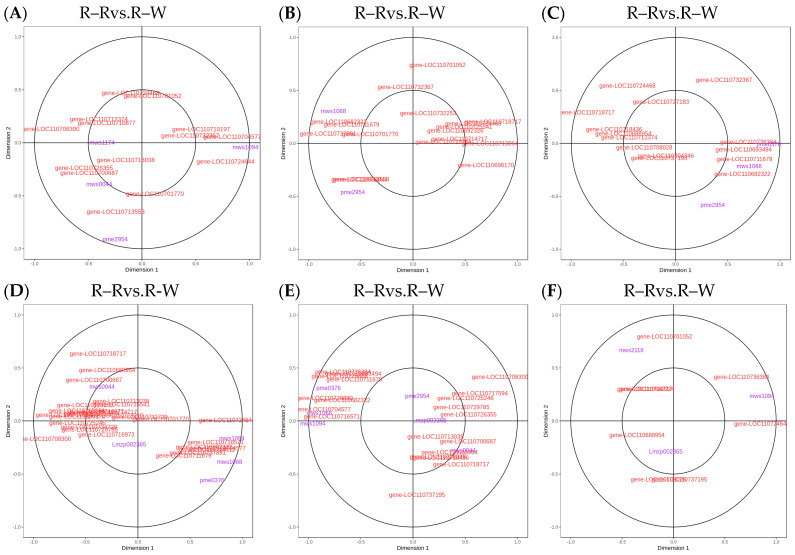
(**A**–**F**): Flavonoid biosynthesis CCA diagrams. Note: The four regions are divided by crosses, and the farther away from the origin and closer to each other in the same region, the higher the correlation.

To understand the differences in seedling synthesis of four different varieties of quinoa, the results of their differential metabolite analyses were combined with the transcriptome differential gene analyses. Mapping of those in the same groups was conducted using a KEGG pathway map, and a histogram was drawn to show the degree of enrichment degree in the pathway with both differential metabolites and genes (Figure 9). Based on the results of the KEGG enrichment analysis, the *p*-value histograms were enriched in flavone and flavonol biosynthesis, flavonoid biosynthesis, and isoflavonoid biosynthesis. The differential ploidy profile using the nine-quadrant plots of gene metabolites (r > 0.8) showed that most genes were consistent with the metabolite differential expression pattern, with upregulated genes and unchanged or downregulated metabolites (Figure S12). The correlation coefficient clustering heatmap (r > 0.8) showed that flavonoids, phenolic acids, and alkaloids accounted for a large proportion of the total content (Figure 10). Correlations between metabolites and genes were represented by network diagrams, and CCA analysis was performed (Figure 11, Tables S23–S34); seven differential genes showed higher correlation coefficient values (r > 0.8) with 10 metabolites, and their interaction networks are shown in Table 7. The seven differentially expressed genes included gene-LOC110700687, gene-LOC110687785, gene-LOC110737891, gene-LOC110736244, gene-LOC110729744, gene-LOC110694588, and gene-LOC110682171, which constitute the major differentially expressed genes in the flavonoid, flavone, flavonol, and isoflavonoid biosynthesis pathways. The 10 metabolites were quercetin-3-O-rutinoside (Rutin), dihydroquercetin (taxifolin), quercetin-3-O-sambubioside, choline, and kaempferol, Quercetin-3-O-(2″-O-xylosyl)rutinoside, choline alfoscerate, N-hydroxytryptamine, 3,4-dihydroxy-L-phenylalanine (L-Dopa), and serotonin. The O2PLS model was used for integration analysis between the two datasets, and all differential genes and metabolites were selected to build the O2PLS model. The loading plot showed that a higher transcriptome weight indicated that the change in this variable perturbed the metabolomics more drastically and that quercetin-3-O-glucoside-7-O-rhamnoside and quercetin-3-O-rutinoside (rutin) were the metabolites that had the greatest impact (Figure S13, Tables S35 and S36).

**Figure 12:**
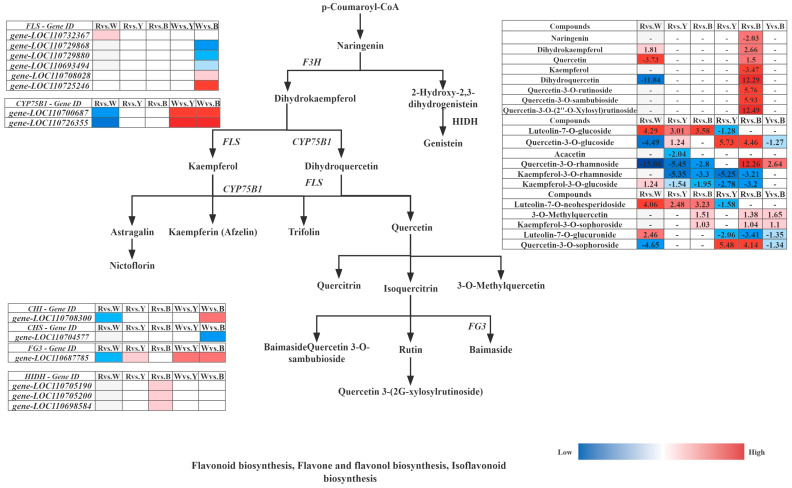
Response mechanisms of flavonoid, flavone, and flavonol biosynthesis, Isoflavonoid biosynthesis in different quinoa seedling cultivars. Note: The box in the pathway represents DEGs and DAMs. Abbreviations are as follows: flavanone 3-hydroxy-lase (F3H), flavonol synthase (FLS), flavonoid 3′-monooxygenase (CYP75B1), flavonol-3-O-glucoside/galactoside glucosyltransferase (FG3).

Furthermore, integrated analysis of the differential synthesis pathways (flavone and flavonol biosynthesis, flavonoid biosynthesis, and isoflavonoid biosynthesis) in six comparative groups of different quinoa cultivars (R–R, R–W, R–Y, and R–B) revealed that R–Rvs.R–W and R–Wvs.R–B had the highest levels of metabolites, and only these two groups were significantly enriched in flavonoid biosynthesis; tryptophan metabolism was only enriched in R–Bvs.R–Y. Cluster analysis of the up and downregulated genes FPKM of key enzyme points in the pathway simultaneously showed that the 14 structural genes contained six FLS (gene-LOC110732367, gene-LOC110729868, gene-LOC110729880, gene-LOC110693494, gene-LOC110708028, and gene-LOC110725246), two CYP75B1 (gene-LOC110700687 and gene-LOC110726355), 1 CHI (gene-LOC110708300), 1 CHS (gene-LOC110704577), 1 FG3 (gene-LOC110687785), and 3 HIDH (gene-LOC110705190, gene-LOC110705200, and gene-LOC110698584), which are the major structural genes in the flavonoid biosynthesis pathway, for flavone and flavonol biosynthesis, and isoflavonoid biosynthesis (Figure 12). Gene-LOC110687785 and gene-LOC110700687 were unmistakably two important differential genes. Overall, R contained more differential metabolites than W, Y, and B, and 29 differential metabolites were detected most frequently in R, and 19 genes were strongly associated with them (Table 8).

## 4. Discussion

Quinoa is an emerging pseudocereal with high nutritional and functional value, as it is rich in nutrients such as minerals, vitamins, polyphenols, and flavonoids and has gluten-free properties [8,31]. It is mainly cultivated for its seeds; however, its seedlings and young leaves are also edible. Differences in the quinoa seedling stage of development have gradually evolved into differences in the grains. However, little is known about the metabolite compositions and molecular mechanisms involved in the biosynthesis of the different quinoa cultivars. In this study, an integrated transcriptomic and metabolite profiling analysis was conducted to identify metabolite compositions and characterize the genes involved in flavonoid, flavone, flavonol, isoflavonoid, tryptophan, glycerophospholipid, and isoquinoline alkaloid biosynthesis in different quinoa seedling cultivars. The metabolite analysis identified 29 differential metabolites and 19 differential genes, which greatly broadened our understanding of the differences that occur during synthesis in the different varieties (red, white, yellow, and black) of quinoa during the seedling stage. Moreover, only 27 metabolites were annotated to the pathways for the 119 flavonoids, and more than half of the flavonoids were glycosides, which are the major forms of flavonoids found in plants; the red and yellow quinoa were previously found to have the highest total flavonoid contents [54,55,56]. Studies have also shown that the phenolic content and composition, flavonoid content, and antioxidant activity differ depending on the quinoa color [23], which means that the total phenolic content depends on the color of the quinoa grains [57]. From the seedling stage, red quinoa has the highest total phenolic content and the most pronounced red leaf color. In the amaranth family, which includes Amaranthus, pigmentation is controlled by betaine in both the leaves and seeds [58,59]. Plant secondary metabolites, including phenolic acids and flavonoids, are biologically active and have various physiological properties, such as antibacterial, antioxidant, anti-inflammatory, antitumor, and anticancer effects [60], which may vary significantly among different species. We have demonstrated that the significantly differential metabolites between the quinoa seedlings include phenolic acids and flavonoids (Table 2). The most abundant flavonoids in quinoa leaves and seeds are flavonol glycosides, as they contain 12 different types, which are composed of kaempferol and quercetin derivatives [61,62]. Further analysis showed that caffeic and ferulic acids and their derivatives are the major phenolic acids, while quercetin and kaempferol and their glycosides are the major flavonoids [61,62,63]. At the metabolomic level, color synthesis begins with the biosynthesis of phenylpropanoid compounds by P-coumaroyl-COA, which is regulated by multiple genes to form the color turning point substance naringenin and opens the flavonoid biosynthesis pathway and affects the flavonoid and flavonol biosynthesis pathways. Naringenin forms dihydrokaempferol under the regulation of naringenin 3-dioxygenase (F3H); meanwhile, under FLS regulation, kaempferol and its derivatives are formed, and quercetin and its derivatives are formed under CYP75AB1 and FLS regulation. Integration of quercetin-3-O-rutinoside (rutin), quercetin-3-O-rhamnoside (quercitrin), quercetin-3-O-sambubioside, quercetin-3-O-glucoside (isoquercitrin), quercetin-3-O-sophoroside (baimaside), quercetin-3-O-(2″-O-xylosyl)rutinoside, 3-O-methylquercetin, and quercetin resulted in the highest accumulation levels in red quinoa and the lowest in white quinoa. The integration of kaempferol (3,5,7,4′-tetrahydroxyflavone), kaempferol-3-O-rhamnoside (afzelin), kaempferol-3-O-glucoside (astragalin), and kaempferol-3-O-sophoroside resulted in the highest accumulation in red quinoa. The accumulation levels for ferulic and caffeic acid were also the highest in red quinoa, and further analysis showed that phenolic acids and flavonoids were highly related to quinoa seedling color development. Betaine has a stronger antioxidant capacity than phenolic compounds, scavenging excess reactive oxygen species (ROS) in plants and humans. The betaine red glycosides accumulate and contribute to color formation in the peel and pulp [23,31], similar to our study, wherein red quinoa had the highest accumulation of betaine red glycosides (betaine-5-O-glucoside), followed by black, white, and yellow quinoa. The transcriptome and structural gene influence were both analyzed using the KEGG color synthesis pathways, which were found to be higher in the R–R than in the R–W, R–Y, and R–B. In particular, both gene-LOC110700687 and gene-LOC110687785 had high expression levels and higher relative expression in red quinoa. In conclusion, the accumulation of flavonoids, phenolic acids, and alkaloids in red quinoa was generally found to be higher with more substrates and enzymes than that in the other three colors, whereas the accumulation of alkaloids in the white quinoa was lower, which may be the reason why the darker the color, the higher the content of flavonoids, phenolic acids, and alkaloids. Our research results provide new insights into the accumulation and coloring mechanisms of flavonoids, phenolic acids, and alkaloids in different colored quinoa seedlings and also provide a theoretical basis for future research.

## 5. Conclusions

The nutritional value and gluten-free nature of quinoa have made it an important food source around the world. However, the differences in the metabolites produced by different colored quinoa cultivars have not yet been thoroughly studied. To address this, the metabolic profiles of four different quinoa cultivars with different colors were effectively discriminated, and their chromatic differences were found to be mainly attributed to flavonoids, phenolic acids, and alkaloid metabolites. The compositions and contents of the flavonoids, phenolic acids, and alkaloid metabolites varied greatly in the different cultivars, and both of them had their own typical compounds. In addition, the expression profiles of the regulatory and structural genes involved in flavonoid biosynthesis, flavonoid and flavonol biosynthesis, and important differential metabolites in phenylpropanoid biosynthesis were assessed. The results showed that the differences in the transcript abundance of key genes could be the reason for the variations in flavonoids, phenolic acids, and alkaloids in the four quinoa seedling cultivars. Overall, this study has improved our understanding of the metabolic mechanisms of quinoa seedlings, will aid in future evaluations of metabolic quality, and will thus create a solid foundation for the future cultivation of high-quality quinoa cultivars.

## Figures and Tables

**Table 1:** Differences in appearance and morphology of four different quinoa seedling cultivars.

Quinoa Color Classification	Plant Height (cm) M ± SD	Stem Thickness (cm) M ± SD	Leaf Area (LAI) (mm^2^) M ± SD	Relative Chlorophyll Content (SPAD)
Red quinoa (R–R)	54.93 ± 1.36 c	1.27 ± 1.01 a	3137.10 ± 22.19 a	49.1
White quinoa (R–W)	69.67 ± 0.59 a	1.10 ± 1.16 bc	2809.21 ± 19.11 ab	44.43
Yellow quinoa (R–Y)	56.55 ± 1.12 b	1.05 ± 1.29 c	2782.80 ± 29.36 c	44.87
Black quinoa (R–B)	65.90 ± 1.57 ab	1.22 ± 2.13 ab	2811.27 ± 16.56 ab	46.67

Note: The mean ± standard deviation is M ± SD; different lowercase letters denote a significant difference at the 0.05 level (*p* < 0.05). Relative chlorophyll content (SPAD) (TYS-4 N).

**Table 2:** Statistical classification of the number of differentially accumulated metabolites.

Group	R–Rvs.R–W	R–Rvs.R–Y	R–Rvs.R–B	R–Wvs.R–Y	R–Wvs.R–B	R–Yvs.R–B
Number of DAMs	169	249	272	250	273	112
Upregulated DAMs	61	76	78	97	111	50
Downregulated DAMs	108	173	194	153	162	62
Number of DAMs annotated by kegg	43	73	83	68	74	47
Amino acid and its derivatives	5	20	19	12	15	10
Phenolic acids	34	48	55	42	47	22
Flavonoid	94	54	56	93	92	30
Alkaloids	10	17	17	12	13	14
Organic acid	6	24	21	14	9	2
Lipids	4	45	66	43	64	17

Note: DAMs represent differentially accumulated metabolites.

**Table 3:** Differential metabolites in different groups of the Venn diagram.

Group	Index	Compounds	Content Comparison	Log_2_FC
R–Rvs.R–W	mws1174	3-O-Acetylpinobanksin	R > B > Y > W	−3.09
R–Rvs.R–Y	mws1078	Anthranilic Acid	R > W > B > Y	−1.13
	mws0051	Acacetin	R > W > B > Y	−2.04
R–Rvs.R–B	mws0047	Apigenin-7-O-neohesperidoside	R > W > Y > B	−1.01
	pme1651	Indole 3-acetic acid (IAA)	Y > W > R > B	−1.24
	mws0853	Sinapyl alcohol	R > W > Y > B	3.02
R–Wvs.R–Y	pme1439	p-Coumaric acid	W > R > B > Y	−1.38
R–Wvs.R–B	NK10253223	2-Amino-3-methoxybenzoic acid	W > R > Y > B	−1.18
R–Yvs.R–B	mws2118	Phloretin-2′-O-glucoside	Y > W > R > B	−1.16

Note: Log_2_FC is the logarithm base 2 of fold change (FC) of the differential metabolite; if log_2_FC is positive, it means upregulation; if log_2_FC is negative, it means downregulation.

**Table 4:** Analysis of differences of significant pathway-related metabolites in quinoa seedling stage of different cultivars.

Index	Compounds	Log_2_FC of Relative Metabolites					
		Rvs.W	Rvs.Y	Rvs.B	Wvs.Y	Wvs.B	Yvs.B
Flavonoid biosynthesis (ko00941)							
mws1094	Aromadendrin (Dihydrokaempferol)	1.81	-	-	−1.66	−2.66	-
pme2954	Quercetin	−3.73	-	-	-	1.50	-
mws1174	3-O-Acetylpinobanksin	−3.09	-	-	-	-	-
mws0044	Dihydroquercetin(Taxifolin)	−1.18	-	-	1.13	1.23	-
Lmzp002365	Hesperetin-7-O-glucoside	-	-	-	3.84	2.82	-
pme0376	Naringenin (5,7,4′-Trihydroxyflavanone)	-	-	-	−1.07	−2.03	-
mws1068	Kaempferol (3,5,7,4′-Tetrahydroxyflavone)	-	-	-	−4.03	−3.47	-
Flavone and flavonol biosynthesis (ko00944)							
pme2954	Quercetin	-	−3.21	−2.24	-	-	-
pme2459	Luteolin-7-O-glucoside (Cynaroside)	-	3.01	3.58	-	-	-
mws0091	Quercetin-3-O-glucoside (Isoquercitrin)	-	1.24	-	-	-	−1.27
mws0051	Acacetin	-	−2.04	-	-	-	-
mws0045	Quercetin-3-O-rhamnoside(Quercitrin)	-	−5.45	-	-	-	-
mws0919	Kaempferol-3-O-rhamnoside (Afzelin)	-	−5.35	−3.30	-	-	-
Lmsn002815	Kaempferol-3-O-rutinoside(Nicotiflorin)	-	1.61	1.43	-	-	-
mws2209	Kaempferol-3-O-glucoside (Astragalin)	-	−1.54	−1.95	-	-	-
mws1068	Kaempferol (3,5,7,4′-Tetrahydroxyflavone)	-	−3.05	−2.50	-	-	-
pmp001079	Luteolin-7-O-neohesperidoside (Lonicerin)	-	2.48	3.23	-	-	-
Lmmn004912	3-O-Methylquercetin	-	-	1.51	-	-	1.65
mws0045	Quercetin-3-O-rhamnoside(Quercitrin)	-	-	−2.80	-	-	2.64
mws0047	Apigenin-7-O-neohesperidoside (Rhoifolin)	-	-	−1.01	-	-	-
Lmyn001269	Kaempferol-3-O-sophoroside	-	-	1.03	-	-	1.10
mws4167	Luteolin-7-O-glucuronide	-	-	-	-	-	−1.35
Lmtp003677	Quercetin-3-O-sophoroside (Baimaside)	-	-	-	-	-	−1.34
Tryptophan metabolism (ko00380)							
pmb0774	N-Hydroxytryptamine	-	−2.28	-	-	−3.53	-
pme2024	Serotonin	-	−2.24	-	-	−3.59	-
Zmtn001624	N-Acetylisatin	-	−2.16	-	-	-	-
pme3083	2-(Formylamino)benzoic acid	-	−1.36	-	-	−1.84	-
mws0005	Tryptamine	-	−3.27	-	-	−4.12	-
Zmtn001464	4,8-Dihydroxyquinoline-2-carboxylic acid	-	3.27	-	-	−1.51	-
mws0677	N-Acetyl-5-hydroxytryptamine	-	−1.18	-	-	-	-
mws1078	Anthranilic Acid	-	−1.13	-	-	-	-
pme1228	5-Hydroxy-L-tryptophan	-	1.07	-	-	−1.01	-
NK10253223	2-Amino-3-methoxybenzoic acid	-	-	-	-	−1.18	-
mws0596	3-Hydroxyanthranilic acid	-	-	-	-	1.15	-
Isoquinoline alkaloid biosynthesis (ko00950)							
pme3827	3,4-Dihydroxy-L-phenylalanine (L-Dopa)	-	-	-	-	-	2.84
pme1002	L-Tyramine	-	-	-	-	-	1.28
Hmgn001653	Protocatechualdehyde	-	-	-	-	-	−1.96
Phosphonate and phosphinate metabolism (ko00440)							
mws2125	Phosphoenolpyruvate	-	-	-	-	-	1.95
pmb0302	2-Aminoethylphosphonate	-	-	-	-	-	1.04

**Table 5:** Statistical classification of the number of DEGs.

Group	R–Rvs.R–W	R–Rvs.R–Y	R–Rvs.R–B	R–Wvs.R–Y	R–Wvs.R–B	R–Yvs.R–B
Number of DEGs	2370	4507	3125	6473	4436	1492
Upregulated DEGs	1318	2592	1760	3547	1949	587
Downregulated DEGs	1052	1915	1365	2926	2487	905

Note: DEGs indicate differentially expressed genes.

**Table 6:** Primer sequences to validate genes.

Quantity	Gene-ID	Gene Description	Primer	5′ to 3′
1	LOC110700687	flavonoid 3′-monooxygenase-like	Forward Primer	TTGACTGACACTGAGATTA
			Reverse Primer	GATTGCGGATTAGTTCTG
2	LOC110726355	flavonoid 3′-monooxygenase-like	Forward Primer	GGAAGAACACAAGGCTAACT
			Reverse Primer	CCTCACCATCACAATTATCTCT
3	LOC110708300	fatty-acid-binding protein 1-like	Forward Primer	CTGATGTCACTGAACCTAA
			Reverse Primer	CCTCCTCAATCCAATACC
4	LOC110687785	anthocyanidin 3-O-glucosyltransferase-like	Forward Primer	TGCTATCTTAATCACTCA
			Reverse Primer	CCATCTTCATCTCTTCTA
5	LOC110737891	fatty-acid-binding protein 3, chloroplastic-like	Forward Primer	CGACTCCTGTTGATGAAT
			Reverse Primer	CAAGCCAAGTTAGAAGAATC
6	LOC110736244	phospholipase A1-Igamma3, chloroplastic-like	Forward Primer	GTCTAATATCCTCTCCTAA
			Reverse Primer	CTTCTTACCGTTCTACTA
7	LOC110729744	carboxylesterase 1-like	Forward Primer	GATTGTTGTGTCTGTTGAG
			Reverse Primer	AGCATCCATAGCATCATC
8	LOC110694588	uncharacterized LOC110694588	Forward Primer	TCCACAAGTTCTGTTCAC
			Reverse Primer	GCAGTAACCGCATCTATA
Internal reference gene	TUB-6	beta-6 tubulin	Forward Primer	TGAGAACGCAGATGAGTGTATG
			Reverse Primer	GAAACGAAGACAGCAAGTGACA

**Table 7:** Correlation analysis between differential genes and differential metabolites of different quinoa cultivars.

Meta ID	Compounds	Accumulation Comparison	Log_2_FC					
			Rvs.W	Rvs.Y	Rvs.B	Wvs.Y	Wvs.B	Yvs.B
pme0376	Naringenin (5,7,4′-Trihydroxyflavanone)	W > R > Y > B	-	-	-	-	−2.03	-
mws1094	Dihydrokaempferol	W > Y > R > B	1.81	-	-	-	2.66	-
pme2954	Quercetin	Y > W > B > R	−3.73	-	-	-	1.50	-
mws1068	Kaempferol	W > Y > B > R	-	-	-	-	−3.47	-
mws0044	Dihydroquercetin(Taxifolin)	B > R > Y > W	−11.84	-	-	-	12.29	-
mws0059	Quercetin-3-O-rutinoside (Rutin)	Y > R > B > W	-	-	-	-	5.76	-
Lmjp002596	Quercetin-3-O-sambubioside	Y > B > R > W	-	-	-	-	5.93	-
Hmcp001618	Quercetin-3-O-(2″-O-Xylosyl)rutinoside	B > Y > R > W	-	-	-	-	12.49	-
pme2459	Luteolin-7-O-glucoside (Cynaroside)	W > B > Y > R	4.29	3.01	3.58	−1.28	-	-
mws0091	Quercetin-3-O-glucoside (Isoquercitrin)	Y > R > B > W	−4.49	1.24	-	5.73	4.46	−1.27
mws0051	Acacetin	B > R > Y > W	-	−2.04	-	-	-	-
mws0045	Quercetin-3-O-rhamnoside(Quercitrin)	R > B > Y > W	−15.06	−5.45	−2.80	-	12.26	2.64
mws0919	Kaempferol-3-O-rhamnoside (Afzelin)	R > W > B > Y	-	−5.35	−3.30	−5.25	−3.21	-
mws2209	Kaempferol-3-O-glucoside (Astragalin)	W > R > Y > B	1.24	−1.54	−1.95	−2.78	−3.20	-
pmp001079	Luteolin-7-O-neohesperidoside	W > B > Y > R	4.06	2.48	3.23	−1.58	-	-
Lmmn004912	3-O-Methylquercetin	B > W > R > Y	-	-	1.51	-	1.38	1.65
Lmyn001269	Kaempferol-3-O-sophoroside	B > R > W > Y	-		1.03	-	1.04	1.10
mws4167	Luteolin-7-O-glucuronide	W > B > Y > R	2.46	-	-	−2.06	−3.41	−1.35
Lmtp003677	Quercetin-3-O-sophoroside (Baimaside)	W > Y > R > B	−4.65	-		5.48	4.14	−1.34
mws1138	Betanin (Betanidin-5-O-glucoside)	R > B > Y > W	−4.06	−1.36	-	2.70	3.92	-
mws0014	Ferulic acid	R > B > W > Y	−2.62	−3.31	−2.13	-	-	1.18
mws2212	Caffeic acid	R > W > Y > B	−1.29	−1.43	−2.28	-	-	-
mws0027	Syringic acid	R > Y > W > B	-	-	−1.48	-	-	-

Note: Log_2_FC is the logarithm base 2 of fold change (FC) of the differential metabolite; if log_2_FC is positive, it means upregulation; if log_2_FC is negative, it means downregulation. “-” indicates no significant difference.

**Table 8:** Correlation network diagram of different quinoa cultivars.

Group	Gene Name	KEGG	Meta Name	Compounds	PCC
R–Rvs.R–W R–Wvs.R–Y R–Wvs.R-B	gene-LOC110700687	K05280 flavonoid 3′-monooxygenase [EC:1.14.14.82]|(RefSeq) flavonoid 3′-monooxygenase-like (A)	mws0059	Quercetin-3-O-rutinoside	0.835
			mws0044	Dihydroquercetin	0.9
			Lmjp002596	Quercetin-3-O-sambubioside	0.8
			Hmcp001618	Quercetin-3-O-(2″-O-Xylosyl)rutinoside	0.83
	gene-LOC110687785	K22794 flavonol-3-O-glucoside/galactoside glucosyltransferase [EC:2.4.1.239 2.4.1.-]|(RefSeq) anthocyanidin 3-O-glucoside 2″-O-glucosyltransferase-like (A)	Hmcp001618	Quercetin-3-O-(2″-O-Xylosyl)rutinoside	0.822
			Lmjp002596	Quercetin-3-O-sambubioside	0.81
R–Rvs.R–Y	gene-LOC110737891	K01859 chalcone isomerase [EC:5.5.1.6]|(RefSeq) probable chalcone--flavonone isomerase 3 (A)	mws1068	Kaempferol (3,5,7,4′-Tetrahydroxyflavone)	0.889
R–Rvs.R-B, R–Wvs.R–Y, R–Wvs.R-B	gene-LOC110736244	K16818 phospholipase A1 [EC:3.1.1.32]|(RefSeq) phospholipase A(1) DAD1, chloroplastic (A)	mws0120	Choline Alfoscerate	−0.8
R–Rvs.R-B, R–Yvs.R-B	gene-LOC110729744	K22097 3-O-acetylpapaveroxine carboxylesterase [EC:3.1.1.105]|(RefSeq) carboxylesterase 1-like (A)	pme3827	3,4-Dihydroxy-L-phenylalanine (L-Dopa)	−0.884
R–Rvs.R–Y, R–Rvs.R-B, R–Wvs.R–Y, R–Wvs.R–B	gene-LOC110694588	K14085 aldehyde dehydrogenase family 7 member A1 [EC:1.2.1.31 1.2.1.8 1.2.1.3]|(RefSeq) aldehyde dehydrogenase family 2 member C4-like (A)	pme2024	Serotonin	0.827
			pmb0774	N-Hydroxytryptamine	0.834
R–Rvs.R–B, R–Wvs.R–B	gene-LOC110682171	K06123 1-acylglycerone phosphate reductase [EC:1.1.1.101]|(RefSeq) hypothetical protein (A)	pmb0484	Choline	−0.822

Note: PCC indicates Pearson’s correlation coefficient.

## Data Availability

The datasets presented in this study can be found in online repositories. The names of the repository/repositories and accession number(s) can be found below: https://www.ncbi.nlm.nih.gov/, accessed on 10 August 2023, PRJNA837037, SRP375030.

## Supplementary Materials

The following supporting information can be downloaded at: https://www.mdpi.com/article/10.3390/metabo13101065/s1, Figure S1: MRM metabolite detection multi peak plot; Figure S2: Total ion flow chart for mass spectrometry analysis of mixed samples; Figure S3: Principal Component Analysis Score Chart; Figure S4: Corresponding group OPLS-DA simulation verification diagram; Figure S5: Differential Metabolite Volcano Map; Figure S6: Venn map of differentially expressed metabolites in different groups;Figure S7: Difference multiple bar chart; Figure S8: Principal component analysis PCA diagram; Figure S9: Differential gene MA map; Figure S10: Venn map of differentially expressed genes in different groups; Figure S11: KEGG Classification Bar Chart; Figure S12: Correlation analysis nine quadrant diagram; Figure S13: O2PLS Model; Table S1: Differences in physiological indicators of quinoa cultivars during seeding stage Specific operating methods; Table S2: All group differential metabolites; Table S3: All metabolites in R–RvsR–W; Table S4: All metabolites in R–RvsR–Y; Table S5: All metabolites in R–RvsR–B; Table S6: All metabolites in R–WvsR–Y; Table S7: All metabolites in R–WvsR–B; Table S8: All metabolites in R–YvsR–B; Table S9: Significantly different metabolites in R–RvsR–W; Table S10: Significantly different metabolites in R–RvsR–Y; Table S11: Significantly different metabolites in R–RvsR–B; Table S12: Significantly different metabolites in R–WvsR–Y; Table S13: Significantly different metabolites in R–WvsR–B; Table S14: Significantly different metabolites in R–YvsR–B; Table S15: A total of differentially significant metabolites in R–R, R–W, R–Y and R–B; Table S16: Number of differential genes in R–RvsR–W; Table S17: Number of differential genes in R–RvsR–Y; Table S18: Number of differential genes in R–RvsR–B; Table S19: Number of differential genes in R–WvsR–Y; Table S20: Number of differential genes in R–WvsR–B; Table S21: Number of differential genes in R–YvsR–B; Table S22: 4368 new gene functions annotated; Table S23: The corresponding genes of the Flavonoid biosynthesis pathway in the CCA diagram are located in R–RvsR–W; Table S24: The corresponding metabolites of the Flavonoid biosynthesis pathway in the CCA diagram are located R–RvsR–W; Table S25: The corresponding genes of the Flavonoid biosynthesis pathway in the CCA diagram are located R–RvsR–Y; Table S26: The corresponding metabolites of the Flavonoid biosynthesis pathway in the CCA diagram are located in R–RvsR–Y; Table S27: The corresponding genes of the Flavonoid biosynthesis pathway in the CCA diagram are located in R–RvsR–B; Table S28: The corresponding metabolites of the Flavonoid biosynthesis pathway in the CCA diagram are located in R–RvsR–B; Table S29: The corresponding genes of the Flavonoid biosynthesis pathway in the CCA diagram are located in R–WvsR–Y; Table S30: The corresponding metabolites of the Flavonoid biosynthesis pathway in the CCA diagram are located in R–WvsR–Y; Table S31: The corresponding genes of the Flavonoid biosynthesis pathway in the CCA diagram are located in R–WvsR–B; Table S32: The corresponding metabolites of the Flavonoid biosynthesis pathway in the CCA diagram are located in R–WvsR–B; Table S33: The corresponding genes of the Flavonoid biosynthesis pathway in the CCA diagram are located in R–YvsR–B; Table S34: The corresponding metabolites of the Flavonoid biosynthesis pathway in the CCA diagram are located in R–YvsR–B; Table S35: O2PLS analysis (transcriptome loading map); Table S36: O2PLS analysis (metabolome loading plot).
