# Supplementary material for: Elucidating the Differentiation Synthesis Mechanisms of Differently Colored Resistance Quinoa Seedings Using Metabolite Profiling and Transcriptome Analysis
Source: Metabolites. 2023 Oct 10;13(10):1065. doi: 10.3390/metabo13101065 (PMC10609267; doi:10.3390/metabo13101065)
Supplement: Supplementary file 1 [file Figure S13.pdf › Figure S13.pdf]

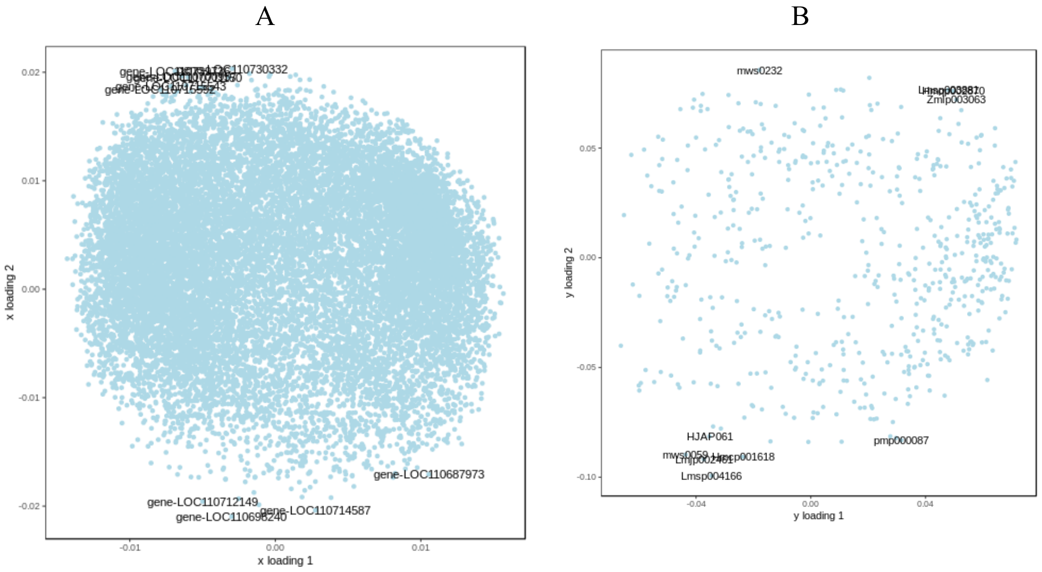

Figure 13 O2PLS Model

Note: The distance of each point to the origin means the magnitude of correlation with another histology, and the top 10 genes and metabolites that have a large impact on another histology are marked in the figure.
