# Supplementary material for: Elucidating the Differentiation Synthesis Mechanisms of Differently Colored Resistance Quinoa Seedings Using Metabolite Profiling and Transcriptome Analysis
Source: Metabolites. 2023 Oct 10;13(10):1065. doi: 10.3390/metabo13101065 (PMC10609267; doi:10.3390/metabo13101065)
Supplement: Supplementary file 1 [file metabolites-13-01065-s001.zip › Figure S1.pdf]

A (N)

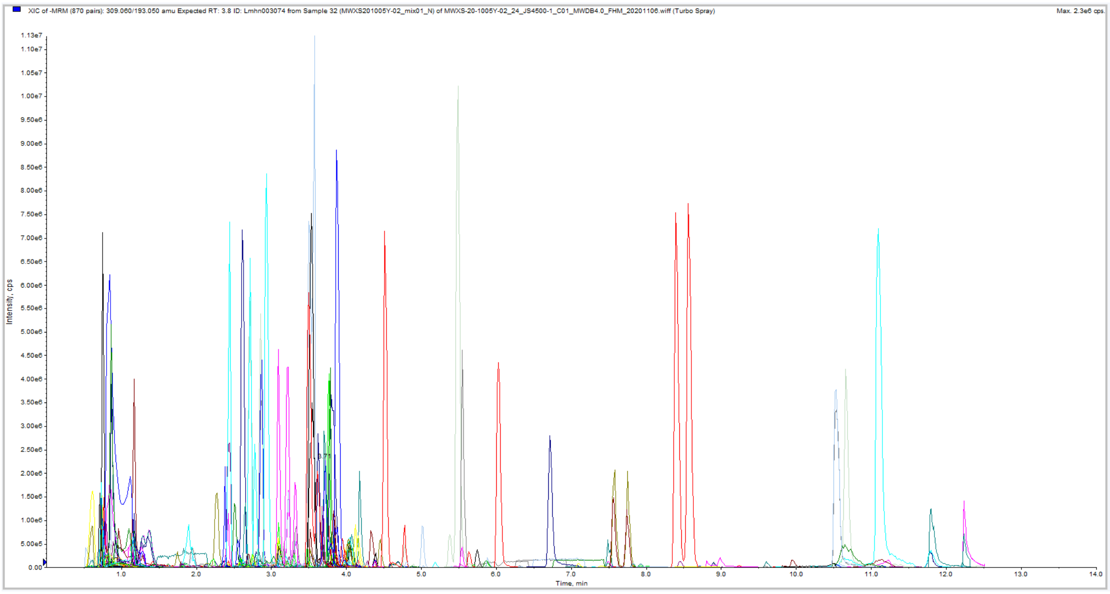

B (P)

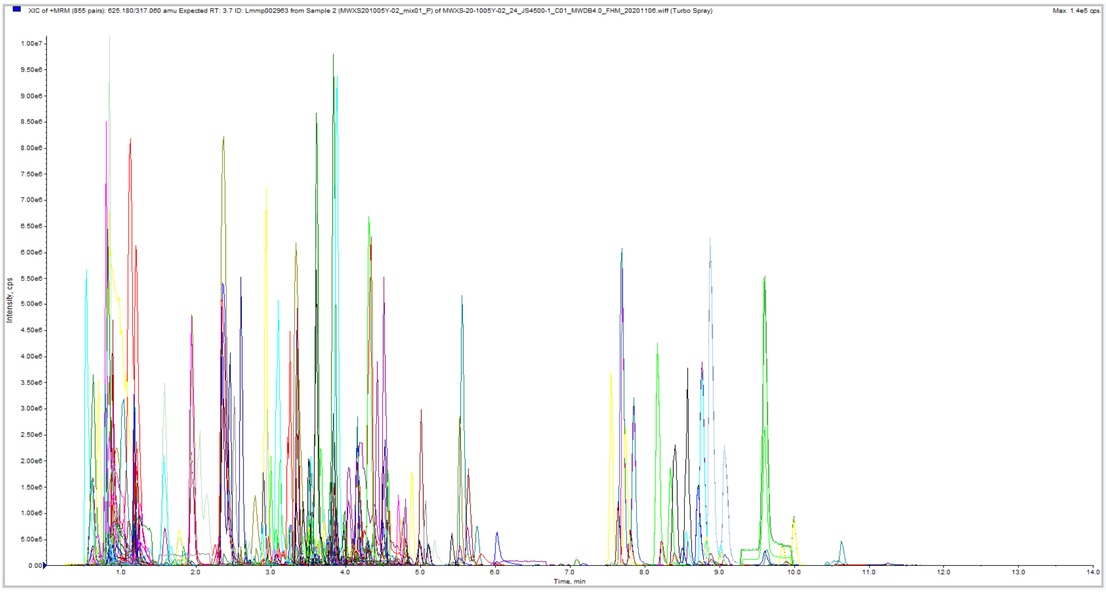

Figure S1 MRM metabolite detection multi peak plot

Note：N represents negative ion mode, P represents positive ion mode.
