# Supplementary material for: Elucidating the Differentiation Synthesis Mechanisms of Differently Colored Resistance Quinoa Seedings Using Metabolite Profiling and Transcriptome Analysis
Source: Metabolites. 2023 Oct 10;13(10):1065. doi: 10.3390/metabo13101065 (PMC10609267; doi:10.3390/metabo13101065)
Supplement: Supplementary file 1 [file metabolites-13-01065-s001.zip › Figure S10.pdf]

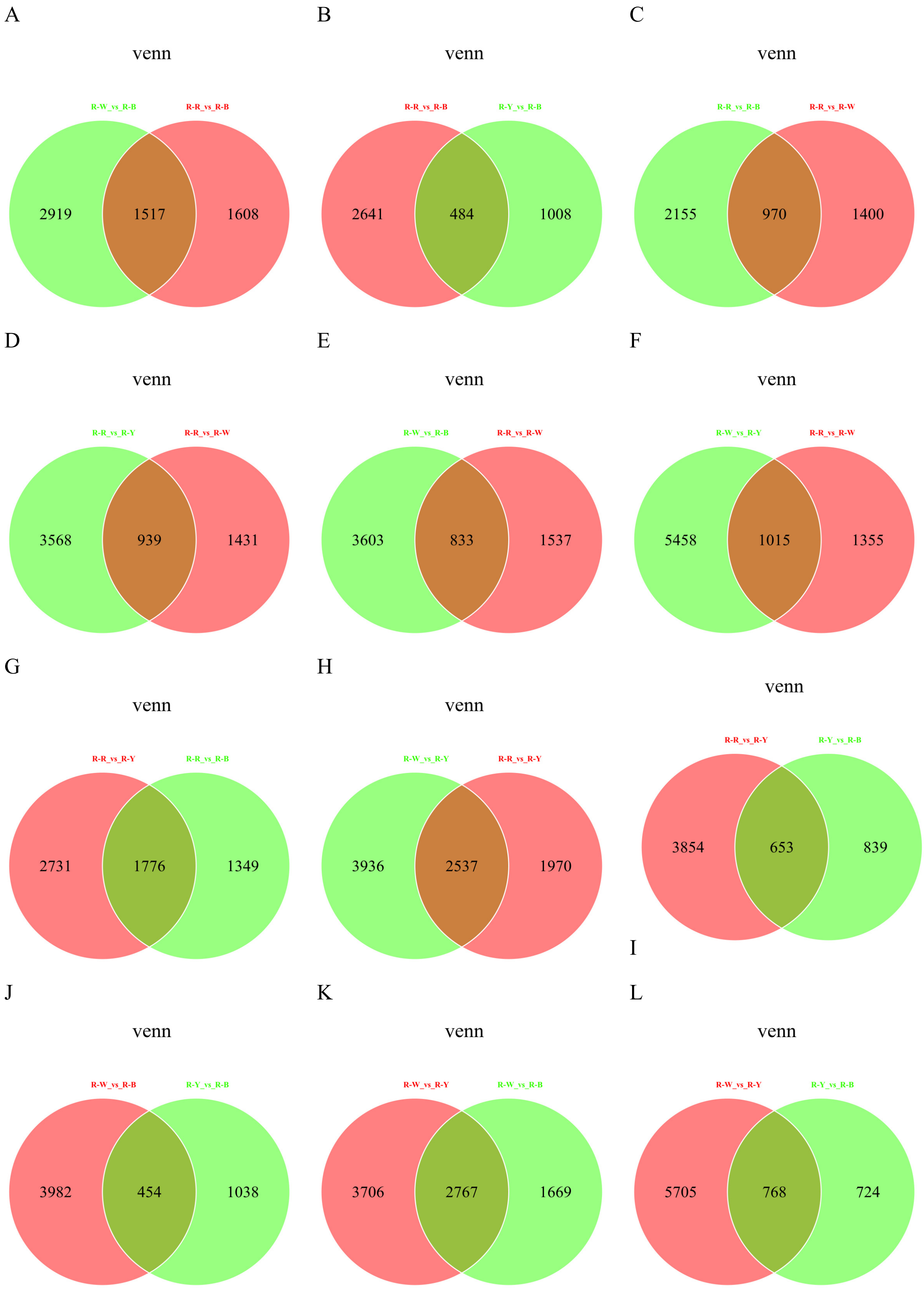

Figure S10 Venn map of differentially expressed genes in different groups

Note: The non-overlapping regions of the Venn map represent differential genes specific to that differential subgroup, and the overlapping regions represent differential genes common to several differential groups that overlap.
