# Supplementary material for: Elucidating the Differentiation Synthesis Mechanisms of Differently Colored Resistance Quinoa Seedings Using Metabolite Profiling and Transcriptome Analysis
Source: Metabolites. 2023 Oct 10;13(10):1065. doi: 10.3390/metabo13101065 (PMC10609267; doi:10.3390/metabo13101065)
Supplement: Supplementary file 1 [file metabolites-13-01065-s001.zip › Figure S11.pdf]

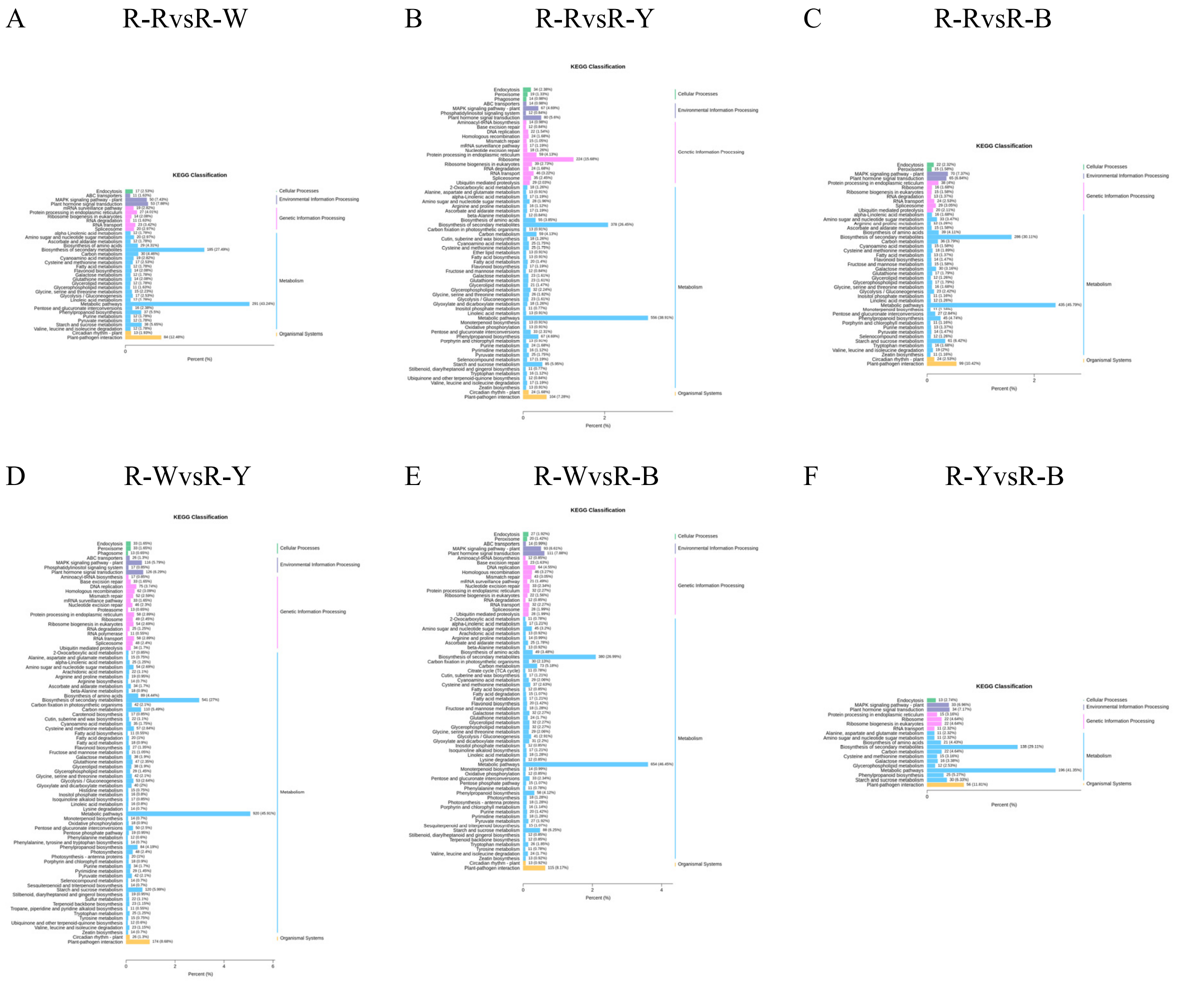

Figure S11 KEGG Classification Bar Chart

Note: The horizontal coordinate indicates the ratio of genes annotated to the pathway to the total number of annotated genes, and the vertical coordinate indicates the name of the KEGG pathway. The labels on the right side of the graph represent the classification to which the KEGG pathway belongs.
