# Supplementary material for: Elucidating the Differentiation Synthesis Mechanisms of Differently Colored Resistance Quinoa Seedings Using Metabolite Profiling and Transcriptome Analysis
Source: Metabolites. 2023 Oct 10;13(10):1065. doi: 10.3390/metabo13101065 (PMC10609267; doi:10.3390/metabo13101065)
Supplement: Supplementary file 1 [file metabolites-13-01065-s001.zip › Figure S12.pdf]

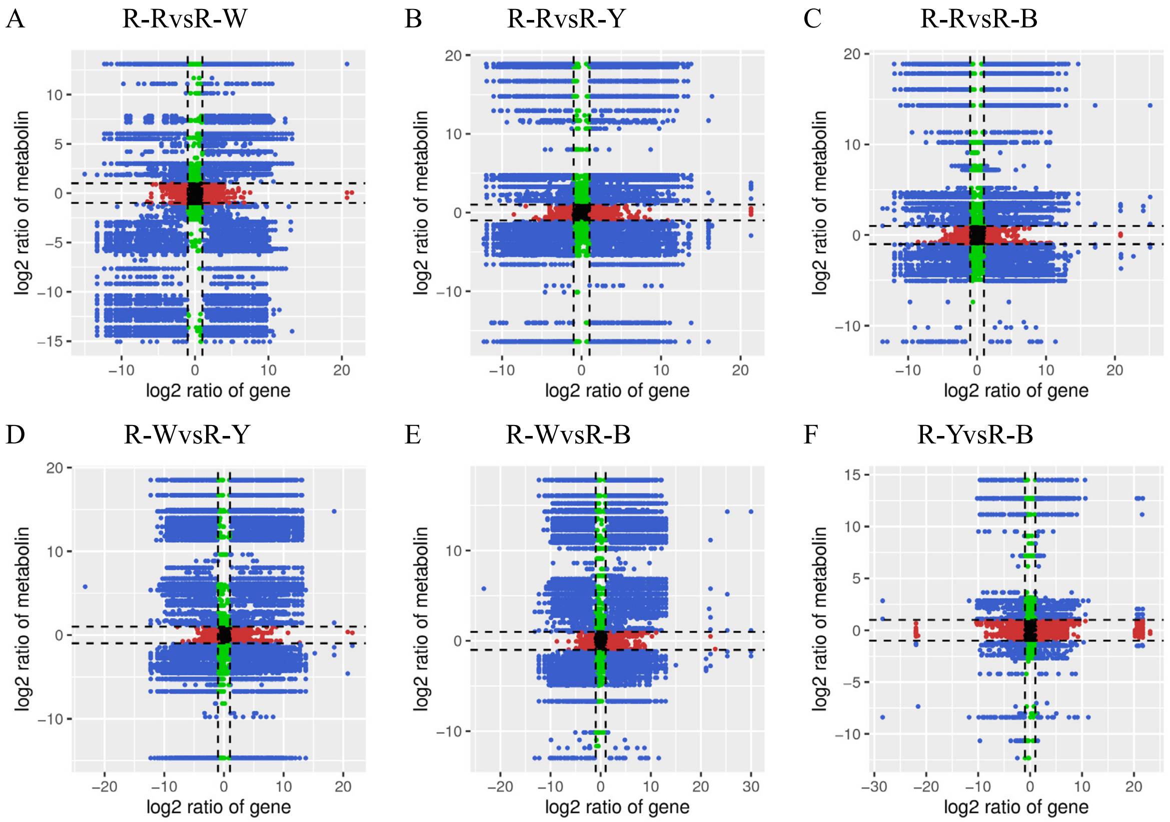

Figure S12 Correlation analysis nine quadrant diagram

Note: From left to right and top to bottom, divided into quadrants 1-9 in order. Quadrant 5 genes and metabolites were not differentially expressed; Quadrant 6,8,9 metabolites were less abundant than genes; Quadrant 1,2,4 metabolites were more abundant than genes; Quadrant 3,7 genes and metabolites were differentially expressed in the same pattern.
