# Supplementary material for: Elucidating the Differentiation Synthesis Mechanisms of Differently Colored Resistance Quinoa Seedings Using Metabolite Profiling and Transcriptome Analysis
Source: Metabolites. 2023 Oct 10;13(10):1065. doi: 10.3390/metabo13101065 (PMC10609267; doi:10.3390/metabo13101065)
Supplement: Supplementary file 1 [file metabolites-13-01065-s001.zip › Figure S2.pdf]

A (N)

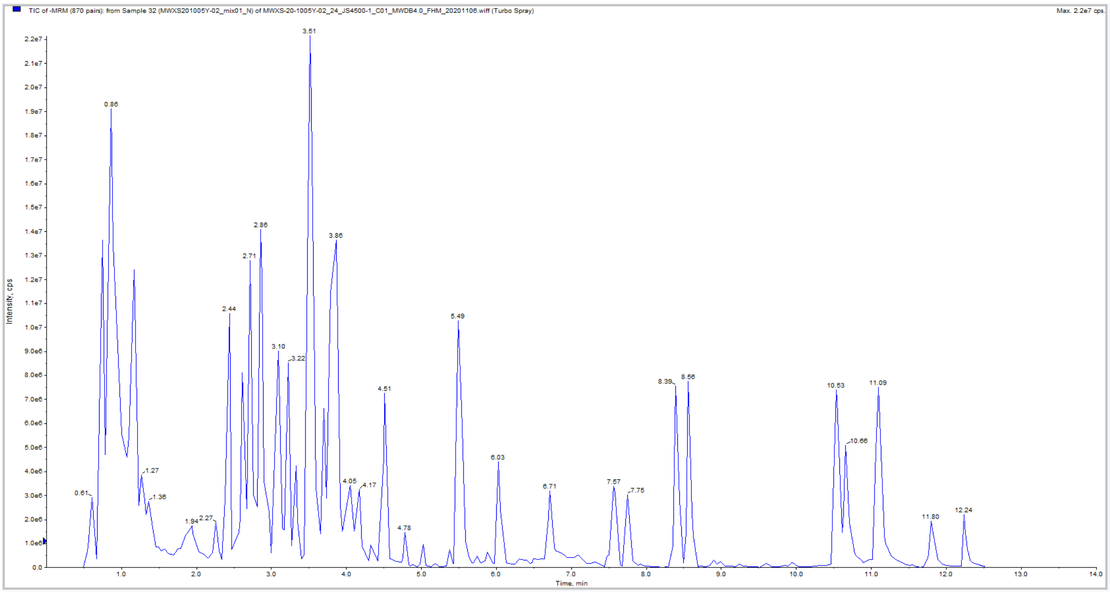

B (N)

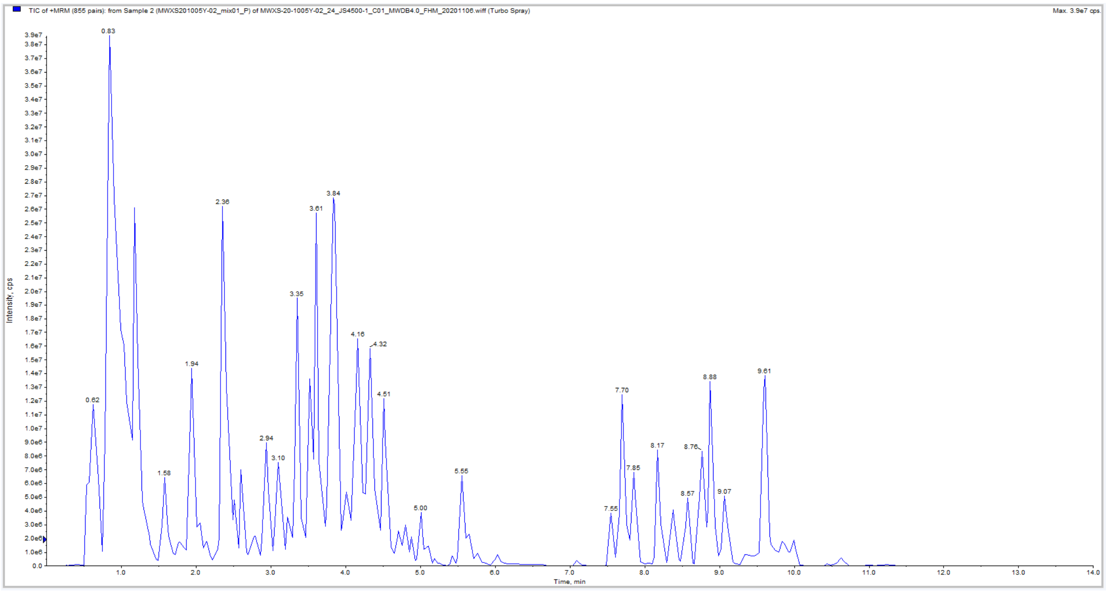

Figure S2 Total ion flow chart for mass spectrometry analysis of mixed samples

Note： N represents negative ion mode, P represents positive ion mode.
