# Supplementary material for: Elucidating the Differentiation Synthesis Mechanisms of Differently Colored Resistance Quinoa Seedings Using Metabolite Profiling and Transcriptome Analysis
Source: Metabolites. 2023 Oct 10;13(10):1065. doi: 10.3390/metabo13101065 (PMC10609267; doi:10.3390/metabo13101065)
Supplement: Supplementary file 1 [file metabolites-13-01065-s001.zip › Figure S3.pdf]

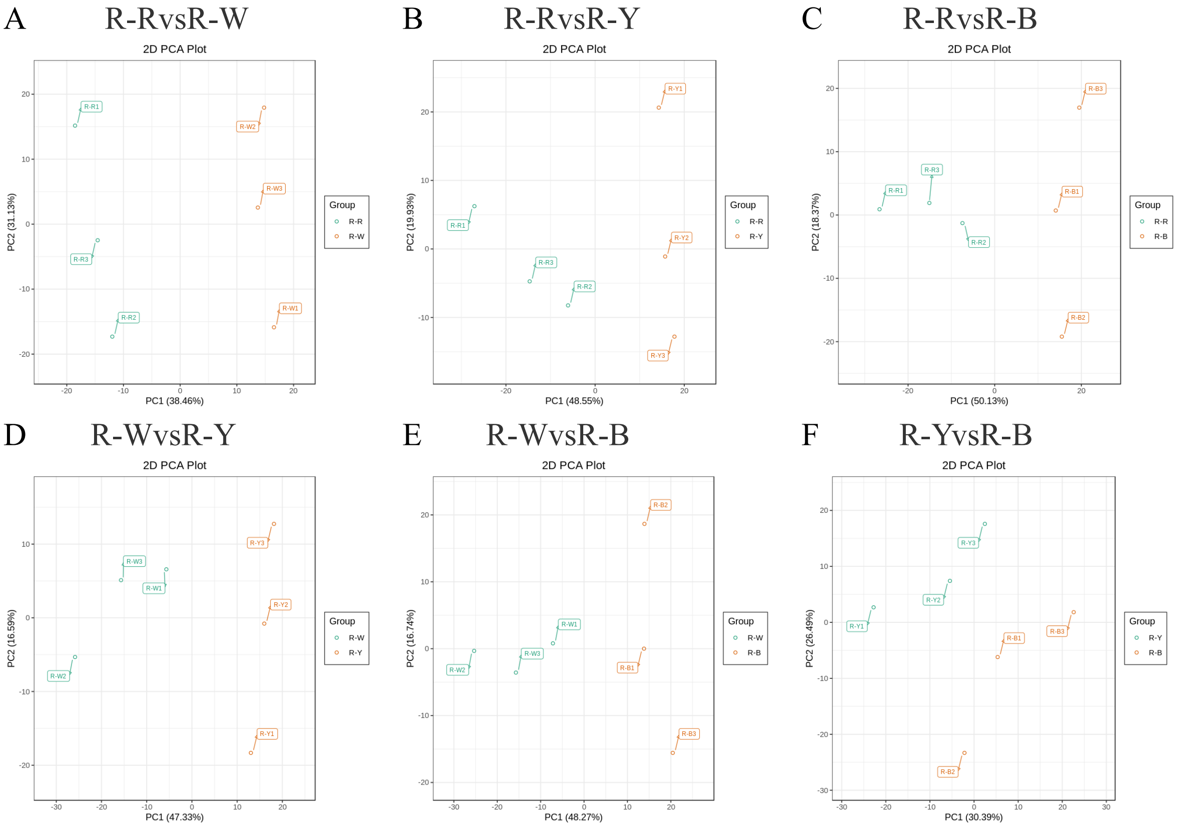

Figure S3 Principal Component Analysis Score Chart

Note: PC1 represents the first principal component, PC2 represents the second principal component, PC3 represents the third principal component, and percentage represents the interpretation rate of this principal component to the dataset; Each point in the figure represents a sample, and samples from the same group are represented by the same color.
