# Supplementary material for: Elucidating the Differentiation Synthesis Mechanisms of Differently Colored Resistance Quinoa Seedings Using Metabolite Profiling and Transcriptome Analysis
Source: Metabolites. 2023 Oct 10;13(10):1065. doi: 10.3390/metabo13101065 (PMC10609267; doi:10.3390/metabo13101065)
Supplement: Supplementary file 1 [file metabolites-13-01065-s001.zip › Figure S4.pdf]

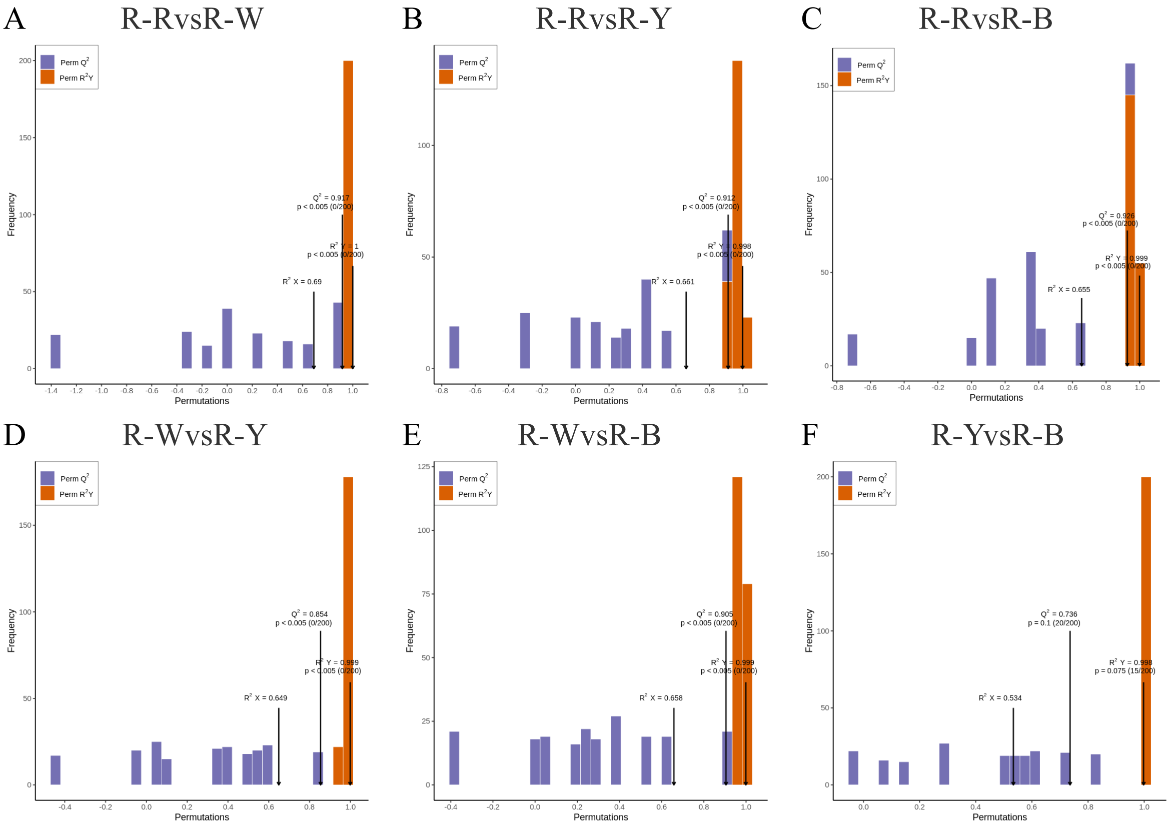

Figure S4 Corresponding group OPLS-DA simulation verification diagram

Note: The prediction parameters of the evaluation model include R^2X, R^2Y, and Q^2, where R^2X and R^2Y represent the explanatory power of the constructed model to the X and Y matrices, and Q^2 represents the predictive ability of the model. The closer these three indicators are to 1, the more stable and reliable the model is. When $Q^2>0.5$, it can be considered an effective model, and when $Q^2>0.9$, it is an excellent model.
