# Supplementary material for: Elucidating the Differentiation Synthesis Mechanisms of Differently Colored Resistance Quinoa Seedings Using Metabolite Profiling and Transcriptome Analysis
Source: Metabolites. 2023 Oct 10;13(10):1065. doi: 10.3390/metabo13101065 (PMC10609267; doi:10.3390/metabo13101065)
Supplement: Supplementary file 1 [file metabolites-13-01065-s001.zip › Figure S5.pdf]

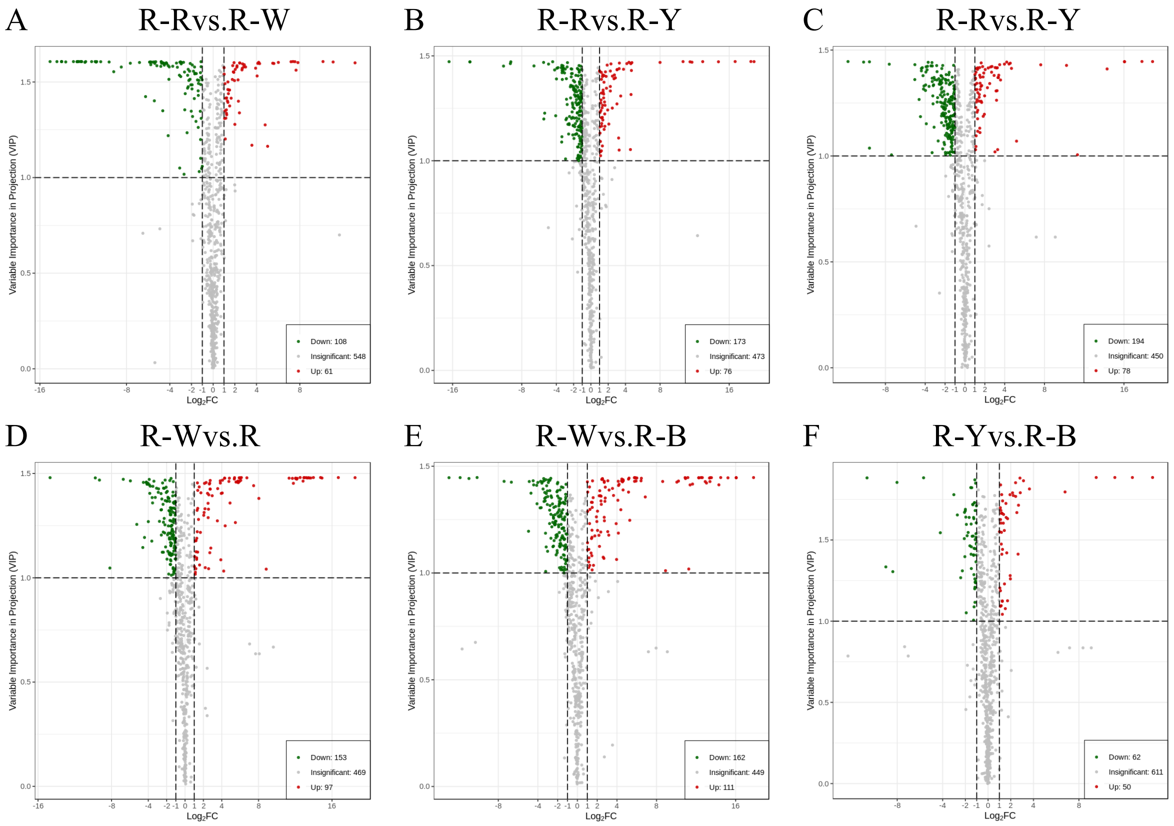

Figure S5 Differential Metabolite Volcano Map

Note: Each point in the volcanic map represents a metabolite, and the abscissa represents the logarithmic value of the quantitative difference multiple of a metabolite in two samples; The vertical axis represents the VIP value. The larger the absolute value of the abscissa, the greater the difference in expression multiples between the two samples; The larger the ordinate value, the more significant the differential expression, and the more reliable the screened differential expression metabolites. The green dots in the figure represent down regulated differentially expressed metabolites, the red dots represent up regulated differentially expressed metabolites, and the gray dots represent metabolites detected but not significantly different.
