# Supplementary material for: Elucidating the Differentiation Synthesis Mechanisms of Differently Colored Resistance Quinoa Seedings Using Metabolite Profiling and Transcriptome Analysis
Source: Metabolites. 2023 Oct 10;13(10):1065. doi: 10.3390/metabo13101065 (PMC10609267; doi:10.3390/metabo13101065)
Supplement: Supplementary file 1 [file metabolites-13-01065-s001.zip › Figure S6.pdf]

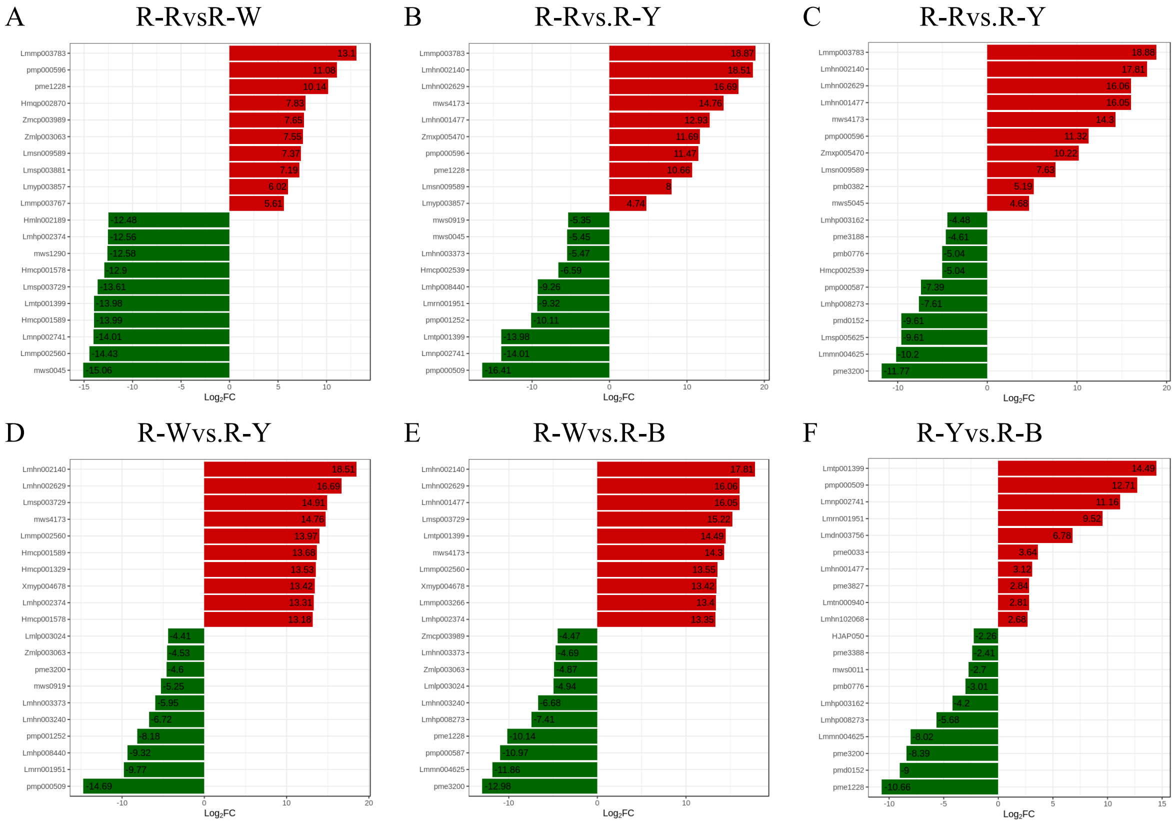

Figure S6 Difference multiple bar chart

Note: The horizontal axis represents the log2FC of differential metabolites, which is the logarithmic value of the difference multiple of differential metabolites with a base of 2. The vertical axis represents the differential metabolites. Red represents upregulated differentially expressed metabolites, while green represents downregulated differentially expressed metabolites.
