# Supplementary material for: Elucidating the Differentiation Synthesis Mechanisms of Differently Colored Resistance Quinoa Seedings Using Metabolite Profiling and Transcriptome Analysis
Source: Metabolites. 2023 Oct 10;13(10):1065. doi: 10.3390/metabo13101065 (PMC10609267; doi:10.3390/metabo13101065)
Supplement: Supplementary file 1 [file metabolites-13-01065-s001.zip › Figure S8.pdf]

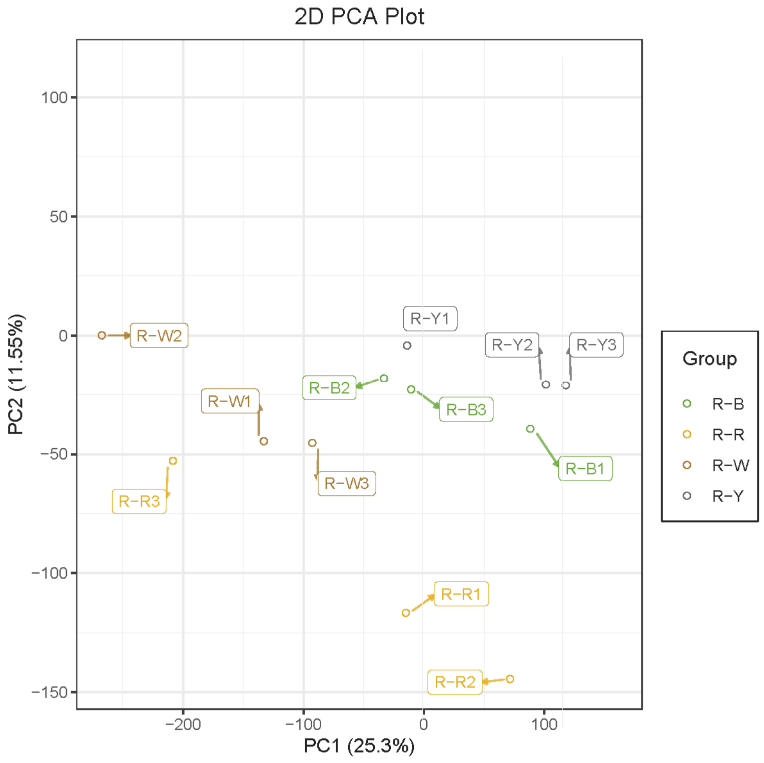

Figure S8 Principal component analysis PCA diagram

Note: PC1 represents the first principal component, PC2 represents the second principal component, and percentage represents the interpretation rate of this principal component to the dataset; Each point in the figure represents a sample, samples from the same group are represented by the same color, and Group represents the grouping.
