# Supplementary material for: Elucidating the Differentiation Synthesis Mechanisms of Differently Colored Resistance Quinoa Seedings Using Metabolite Profiling and Transcriptome Analysis
Source: Metabolites. 2023 Oct 10;13(10):1065. doi: 10.3390/metabo13101065 (PMC10609267; doi:10.3390/metabo13101065)
Supplement: Supplementary file 1 [file metabolites-13-01065-s001.zip › Figure S9.pdf]

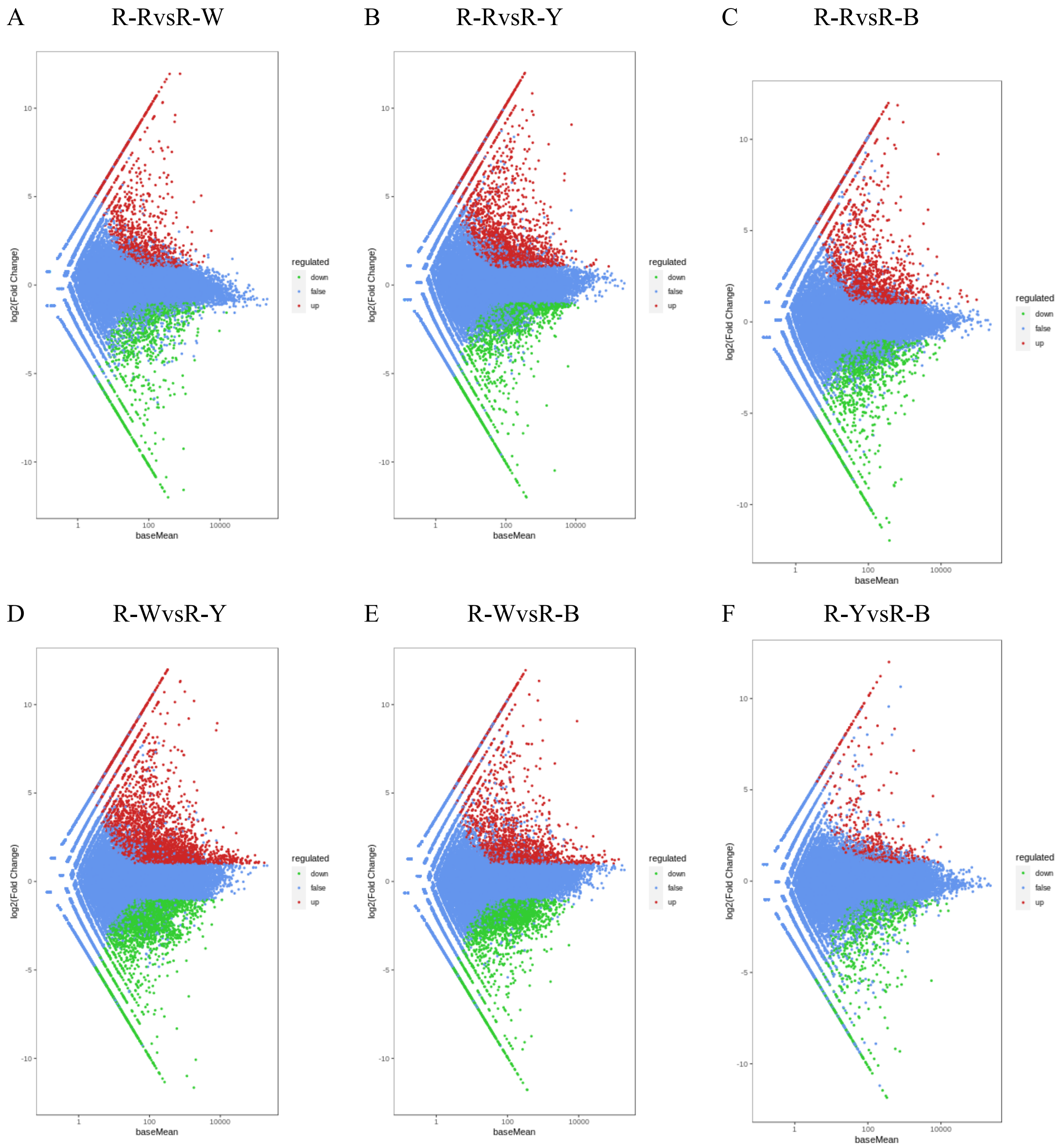

Figure S9 Differential gene MA map

Note: The vertical axis represents the log2FC value, representing the logarithmic value of the difference multiple (log2 Fold_Change); The horizontal axis represents the average gene expression level between the two samples; The red dots represent an upregulation of gene expression, the green dots represent a downregulation, and the blue dots indicate no significant difference in gene expression.
