# Supplementary material for: Elucidating the Differentiation Synthesis Mechanisms of Differently Colored Resistance Quinoa Seedings Using Metabolite Profiling and Transcriptome Analysis
Source: Metabolites. 2023 Oct 10;13(10):1065. doi: 10.3390/metabo13101065 (PMC10609267; doi:10.3390/metabo13101065)
Supplement: Supplementary file 1 [file metabolites-13-01065-s001.zip › Table S1.pdf]

The physiological and biochemical indicators of the difference between insect resistance and insect susceptibility of quinoa were as follows:

(1) Total alkaloids (total alkaloid content kit-spectrophotometric method): the harvested leaves were dried at 60°C, crushed, and sieved. Next, 0.05 g of the sieved sample was placed into a 2-mL EP tube and 1 mL of extraction solution was added. The extraction was then shaken at room temperature for 30 min and sonicated for a 30-min period consisting of intervals whereby the extraction was taken out and shaken for 1 min every 3 min, and then sonicated again. Finally, the extract was made up to 1 mL, centrifuged at 4000 rpm at room temperature for 10 min, and the supernatant was extracted for further analysis.

(2) Flavonoids (plant flavonoids assay kit - colorimetric method): the harvested leaves were dried to a constant weight, crushed, and sieved. The sieved leaf fragments weighed approximately 0.02 g. Next, 2 mL of 60% ethanol was added, and the extraction was shaken at 60°C for 2 h and centrifuged at 10,000 g for 10 min at room temperature. The supernatant was then extracted and measured.

(3) Soluble sugar (plant soluble sugar detection kit anthrone colorimetry): Leaves were weighed to 0.1 g, before being grinded into a homogenate using 1 mL distilled water. This homogenate was then soaked in boiling water for 10 min and left to cool before being centrifuged at 4000 rpm for 10 min at room temperature. The supernatant was diluted 10 x using distilled water and then left on a shaker until further use.

(4) Total amino acid (T-AA; total amino acid test kit colorimetric method): after accurately weighing the tissue sample, nine times the normal saline was added according to the weight volume ratio to make a 10% homogenate. This was then subjected to centrifugation at 3,500 rpm for 10 min, and the supernatant was extracted for analysis. In addition, 10% of the homogenate supernatant was taken for protein determination.

(5) Total phenols (total plant phenols test box spectrophotometry): Approximately 0.1 g of leaf powder was weighed out, to which 2ml PBS buffer solution was added. The solution was vortexed for 2–3 minutes and then placed in a water bath set to 60°C for

30 min. Finally, the solution was centrifuged at 400 rpm for 10 min, and the supernatant was extracted for further analysis.
